# Metal nanoparticle hybrid hydrogels: the state-of-the-art of combining hard and soft materials to promote wound healing

**DOI:** 10.7150/thno.91829

**Published:** 2024-01-27

**Authors:** Yuxiang Wang, Mengya Zhang, Zhenzhen Yan, Shizhao Ji, Shichu Xiao, Jie Gao

**Affiliations:** 1 Department of Burn Surgery, The First Affiliated Hospital of Naval Medical University, Shanghai, 200433, People's Republic of China.; 2 Changhai Clinical Research Unit, The First Affiliated Hospital of Naval Medical University, Shanghai, 200433, People's Republic of China.; 3 Shanghai Key Laboratory of Nautical Medicine and Translation of Drugs and Medical Devices, Shanghai, 200433, People's Republic of China.

**Keywords:** Metal nanoparticles, Hydrogels, Wound healing, Skin tissue engineering, Biomaterials

## Abstract

Wounds represent a grave affliction that profoundly impacts human well-being. Establishing barriers, preventing infections, and providing a conducive microenvironment constitute the crux of wound therapy. Hydrogel, a polymer with an intricate three-dimensional lattice, serves as a potent tool in erecting physical barriers and nurturing an environment conducive to wound healing. This enables effective control over exudation, hemostasis, accelerated wound closure, and diminished scar formation. As a result, hydrogels have gained extensive traction in the realm of wound treatment. Metallic nanoparticle carriers, characterized by their multifaceted responses encompassing acoustics, optics, and electronics, have demonstrated efficacy in wound management. Nevertheless, these carriers encounter challenges associated with swift clearance and nonuniform effectiveness. The hybridization of metallic nanoparticle carriers with hydrogels overcomes the shortcomings inherent in metallic nanoparticle-based wound therapy. This amalgamation not only addresses the limitations but also augments the mechanical robustness of hydrogels. It confers upon them attributes such as environmental responsiveness and multifunctionality, thereby synergizing strengths and compensating for weaknesses. This integration culminates in the precise and intelligent management of wounds. This review encapsulates the structural classifications, design strategies, therapeutic applications, and underlying mechanisms of metal nanoparticle hybrid hydrogels in the context of acute and chronic wound treatment. The discourse delves into the generation of novel or enhanced attributes arising from hybridization and how the current paradigm of wound therapy leverages these attributes. Amidst this continually evolving frontier, the potential of metal nanoparticle hybrid hydrogels to revolutionize wound treatment is underscored.

## 1. Introduction

A wound refers to the impairment of the skin's barrier function caused by external forces or internal diseases. Based on the healing timeline, wounds can be classified as acute or chronic 1. Burn injuries and diabetic foot ulcers (DFUs) stand as two archetypal instances of compromised skin barrier function 1. Burns are common acute wounds, signifying the most severe forms of damage and trauma. Based on data from the World Health Organization, it has been determined that approximately 180,000 individuals experience fatal outcomes due to burns on a yearly basis 2. This occurrence is predominantly prevalent in countries with low to middle income levels. In the specific case of India, more than 1 million individuals suffer from moderate to severe burns annually. Similarly, approximately 173,000 Bangladeshi children experience moderate to severe burns each year. The financial burden associated with providing direct care for burns varies significantly, but generally leans towards being costly. A systematic review conducted in 2014 revealed an average total healthcare cost per burn patient of US$ 88,218 3. Concurrently, chronic wounds induced by diabetes present a prominent challenge in wound management and stand as a leading cause of mortality in diabetic patients 4. Presently, the global diabetic population stands at 537 million and is projected to reach 783 million by 2045 4. Approximately 25% of diabetic patients are at risk of chronic nonhealing wounds, exemplified by diabetic foot ulcers (DFUs) 5. In addressing the impediments to wound healing and infection, the swift establishment of a provisional skin barrier proves pivotal. Traditional wound dressings predominantly encompass regular gauze and petrolatum-infused gauze. While these materials shield wounds from contamination, their efficacy in fluid absorption and infection resistance remains limited. Additionally, they fail to provide the requisite mildly moist environment necessary for optimal wound healing. Moreover, they necessitate frequent replacement within a short span, inflicting considerable discomfort upon patients 6,7. Biological materials such as allogeneic skin, xenogeneic skin, and amnion serve as ideal temporary wound coverings. These materials foster a mildly moist wound environment, accelerate wound healing, and alleviate the anguish associated with frequent dressing changes. However, they are encumbered by constraints, including insufficient skin sources, inconvenient preservation and usage, and suboptimal infection resistance 8,9. Hence, contemporary wound dressings should inherently embody the following attributes: provision of a mildly moist wound environment; reduction of wound exudation; potent infection resistance; diminished dressing change frequency; and alleviated patient discomfort.

Hydrogels possess distinct physicochemical and mechanical properties, including water absorption and retention, biodegradability, and tunable viscoelasticity, thereby providing effective barriers that absorb wound exudates, reduce wound moisture evaporation and prevent bacterial infiltration 10-13. Thus, hydrogels foster a microenvironment conducive to healing 14-17. Furthermore, hydrogels, as extracellular matrix (ECM), are capable of supporting and enhancing cell migration and proliferation to facilitate tissue repair 16,18-20; as a result, hydrogels are composed of a soft and hydrophilic three-dimensional network of crosslinked polymers 21-23.

Given these attributes, hydrogels have emerged as promising novel wound healing dressings 14,24. However, additional limitations in wound dressings include singular functionality, inferior mechanical strength, susceptibility to physiological degradation, limited resistance to multidrug-resistant bacteria, which fails to meet the requirements of processing and shaping, frequent and everlasting exposure to bacteria and free mobility around wound tissue. Hence, these challenges highlight the importance of developing modifications and adaptations of hydrogels as wound dressings in terms of structure and function.

Nanomaterials have a wide range of biological activities, of which metal nanoparticles are outstanding representatives 25-30. Metal nanoparticles are three-dimensional crystalline materials with diameters ranging from 1 to 100 nm. They possess a high surface area-to-volume ratio and come in various sizes, shapes, and physicochemical activities 31. Therefore, they serve as drug delivery vehicles and carriers owing to their strong stability, high loading capacity, specific targeting, and trigger release capabilities. Metal nanoparticles are notably extended to be utilized in wound healing as a result of reducing mitochondrial membrane potential, triggering neutrophil apoptosis and consequently reducing cytokine production 6. Furthermore, photothermal effects induced by metal nanoparticles stimulate the proliferation and migration of epidermal cells and fibroblasts, expediting wound healing 32 and eliminating bacteria 33,34. Moreover, metal nanoparticles inhibit microorganisms through actions such as penetrating bacterial membranes, inducing intracellular damage, and generating intracellular reactive oxygen species (ROS), thus exhibiting superior antibacterial performance 33. However, metal nanoparticles exhibit certain toxicity toward normal tissue cells 34, while they are easily oxidized or degraded due to oxygen or pH fluctuations 35 and cleared by macrophages 32. Hence, modifications and upgrades to metal nanoparticles in wound treatment are imperative to address high toxicity, poor stability, and short circulation.

Metal nanoparticle hybrid hydrogels (MNHHs) represent a significant advancement in biomedical materials, resulting from the fusion of metal nanoparticles with hydrogels. This innovative hybrid hydrogel not only enhances the stability and circulation time of metal nanoparticles, thereby mitigating their toxicity, but also exhibits responsiveness to a wide range of physicochemical stimuli, including acoustic, light, and electric stimuli. Furthermore, the distribution of metal nanoparticles within the hydrogel enhances its physical and mechanical properties, such as stretchability and rheology, effectively meeting the diverse requirements for wound healing. The versatile nature of MNHHs has led to their widespread application in various biomedical contexts, encompassing antimicrobial wound dressings 36, environmentally responsive therapies 37, tissue engineering scaffolds 38, and biosensing detection 39-41. Nevertheless, the application of MNHHs in wound treatment is not without challenges. Current issues include the environmental impact of large-scale production, the high cost hindering clinical adoption, and the potential long-term toxic effects of accumulated metal nanoparticles in the human body. In this emerging field of MNHH, although there have been some reviews discussing the application progress of specific types of MNHH (e.g., silver, ZnO), to our knowledge, there is currently no comprehensive and systematic overview of the types, design strategies, and mechanisms of action of MNHH in wound healing. Hence, there is a need for a comprehensive summary and analysis of the research progress of MNHH, its potential applications, and challenges in the field of wound healing.

In this review, we introduce common design strategies, research advancements, and challenges related to MNHH for wound healing applications. We briefly explain the gelation mechanism and loading methods of MNHH, outline their functional characteristics, and comprehensively elaborate on the mechanisms by which MNHH promotes wound healing. Through this review, we present the latest progress, challenges, and prospects of MNHH, which may inspire the design of advanced composite hydrogel materials and provide valuable insights for the clinical translation of these emerging materials for wound healing purposes.

## 2. The design strategies of MNHH

Hydrogels are crosslinked through physical mechanisms such as ionic bonds or chemical methods such as Schiff's base bonds. Metal nanoparticles can be loaded into hydrogels using various approaches. For instance, metal nanoparticle precursor ions can be adsorbed onto the polymer matrix and then reduced in situ to form nanoparticles, thus integrating them into the hydrogel 42-45. Although the metal nanoparticles are uniformly dispersed within the hydrogel matrix using this approach, it is typically employed for polymer systems or reactions involving reduction. Alternatively, colloidal suspensions containing metal nanoparticles can be mixed with the polymer solution before gelation, effectively immobilizing the nanoparticles within the hydrogel matrix 46,47. The blending process is straightforward and results in the formation of stable networks. However, initiation of polymerization requires the use of an initiator or an external system, such as a photoinitiator with light, HRP (Horse radish peroxidase) with H_2_O_2_, or even temperature. Finally, the polymer solution can be initially gelled into the primary hydrogel structure, and subsequently, metal nanoparticles can be incorporated through covalent chelation, physical embedding, or electrostatic interactions after gelation 48-50, representing a comprehensive strategy for the formation of a double or multi-network. The majority of the formed networks may be reversible, allowing them to adapt to the extracellular matrix (ECM) due to the transformable bonding between polymer chains and metal nanoparticles. Notably, metal nanoparticles can serve as crosslinking centers through various mechanisms, significantly enhancing the mechanical and physicochemical properties of the hydrogel. Metal nanoparticles act as physical crosslinkers by forming interparticle interactions and increasing the entanglement of polymer chains. Additionally, modified or metal nanoparticles themselves possess functional groups on their surfaces that can react with the polymer chains, leading to the formation of covalent bonds. For example, gold nanoparticles tend to bind and cap to sulfur groups due to the strong ionic and covalent bonds of Au-S. Leveraging the dynamic exchangeability of Au-S, thiol end group ligands modified polymers can be attached to thiolated Au nanoparticles, such as polyethylene glycol (PEG) 51-53. Initially, the focus of metal nanoparticle hybrid hydrogels (MNHH) was primarily on enhancing the mechanical strength of composite hydrogel materials. Today, MNHHs exhibit a wider range of functionalities. First, the multi-responsive nature of metal nanoparticles—responsive to stimuli such as sound, light, and electricity—imparts the hydrogel with precise disease diagnostics and treatment capabilities. Second, the environmental sensitivity of hydrogel structures alters the optical, chemical, or electronic properties of embedded metal nanoparticles. Furthermore, the distribution of metal nanoparticles within the hydrogel's spatial network structure leads to the creation of materials with super-performance or composites featuring enhanced modified nanocarriers. For instance, hydrogels loaded with metal nanoparticles can be employed to develop multifunctional wound dressings that combat drug-resistant pathogens and promote wound healing 34,54-56. They can also serve as components of smart wound dressing systems 35,57,58, providing scaffolds for cell differentiation, manipulation, and proliferation, integrated with real-time monitoring and on-demand therapy to overcome the limitations of traditional wound management 39,41. As such, MNHHs exhibit enhanced mechanical properties and multifunctionality, offering effective interventions for wound management. This advancement holds promise for improving wound healing processes and patient outcomes.

## 3. The mechanisms of the enhancement functions of MNHH

### 3.1. How metal nanoparticles improve the properties of hydrogels

Metal nanoparticles can indeed enhance the mechanical strength and stability of hydrogels. The integration of metal nanoparticles as structural components promotes the physical or chemical crosslinking of hydrogels, leading to improved mechanical and rheological properties. For instance, research has demonstrated the use of TiO_2_ nanoparticles to enhance the physicochemical and rheological properties of hydrogels 59. The formation of localized microstructures and permeation leads to changes in the gel structure, resulting in an increase in viscoelastic modulus and a decrease in frequency dependency, resembling more solid-like behavior 59. Furthermore, a composite material, gelatin/CS/Ag, was obtained by in situ reduction of silver nanoparticles using gelatin as a reducing and stabilizing agent mixed with chitosan 60. This composite hydrogel exhibited excellent mechanical properties, water absorption, and moisture retention, along with good biocompatibility 60. In another example, cobalt nanoparticles were crosslinked into a poly (hydroxyethyl methacrylate) (pHEMA) hydrogel, resulting in improved loading efficiency and reduced leaching effects. The resulting composite material demonstrated muscle-like movement and flexibility 61. The incorporation of metal nanoparticles can significantly enhance the mechanical properties and overall performance of hydrogels, making them promising materials for various biomedical applications.

The self-healing ability of hydrogels often arises from reversible noncovalent interactions between polymer chains, and interactions between polymer chains and metal ions can also drive the self-healing of composite materials 62. As colloids in solution, the surface ligands, morphology, and size of metal nanoparticles determine their interfacial interactions. Metal nanoparticle-loaded hydrogels can utilize the self-assembly behavior and optical activity of metal nanoparticles for self-repair. For instance, research has indicated that azobenzene-functionalized gold nanoparticles can undergo photoisomerization under ultraviolet light. Combining this nanomaterial with a hydrogel imparts the hydrogel with the ability to self-heal under photothermal stimulation 63. Additionally, the coordination between polymers and metal surfaces typically remains unchanged due to local environmental ion variations 64. Therefore, the introduction of metal nanoparticles can enhance the robustness of hydrogel self-healing performance. In a study, gelatin modified with dopamine was used as a biomimetic template to synthesize a composite hydrogel material called Gel-DA @ Ag nanoparticles. This material exhibited good mechanical properties, injectability, and excellent self-healing capabilities 65. The incorporation of metal nanoparticles into hydrogels not only enhances their mechanical and responsive properties but also adds a self-healing dimension to their functionalities, making them even more promising for advanced biomedical applications.

Furthermore, metal nanoparticle hybrid hydrogels (MNHH) have the potential to enhance the drug delivery system within hydrogels and refine the form of drug release. Due to the propensity of hydrogels to degrade in wound environments and their inherent mechanical instability, the sustained stability of their drug delivery systems is compromised. The incorporation of metal nanoparticles as structural components induces physical cross-linking, thereby improving the mechanical properties of hydrogels, enhancing their stability as drug delivery systems, and prolonging the drug release duration. For instance, the integration of Ag nanoparticles end-capped with benzotriazole maleimide into furfural-modified gelatin, alongside the addition of chondroitin sulfate (CS), yields mechanically stable MNHH 49. Although the introduction of Ag nanoparticles might diminish the overall loading capacity of hydrogel systems for hydrophilic drugs, the augmentation of mechanical stability leads to an extended drug release period 49,66. Additionally, metal nanoparticles endowed within MNHH with active or passive stimulus-responsive capabilities, encompassing responsiveness to external photothermal stimulation and localized environmental cues at wound sites, facilitate precise, intelligent, and targeted drug release. The local biological stimuli at wound sites generally encompass pH, temperature, and ion concentration. For instance, when the metal nanoparticles within MNHH are consistently exposed to specific wavelengths of light, their temperature rapidly rises, thus effectuating photothermal therapeutic (PTT) effects. Simultaneously, the incorporation of metal nanoparticles into MNHH to realize PTT can be employed to modulate drug release through the thermal-responsive deformation of hydrogels 67. For instance, composite hydrogels containing polydopamine (PDA)-coated gold nanoparticles (Au NPs) and doxorubicin (DOX) firmly anchored within the hydrogel matrix through PDA and amide interactions can achieve PTT and controlled DOX release upon near-infrared (NIR) light irradiation 68. Similarly, MNHH can achieve drug modulation akin to photothermal effects through magneto-thermal responses 69.

In addition to enhancing and refining the inherent properties of hydrogels, metal nanoparticles can also bestow upon them entirely novel characteristics, including environmental responsiveness, biomarker monitoring, and imaging capabilities. These attributes are not independent but rather interconnected and mutually influencing, collectively propelling the application of composite materials in wound treatment. Environmentally responsive hydrogels, also known as smart hydrogels, are hydrogel materials that exhibit the ability to perceive intrinsic or extrinsic environmental stimuli and enact moderate responses accordingly. The introduction of metal nanoparticles grants hydrogels an entirely new environmental responsiveness, encompassing light sensitivity, magnetic field sensitivity, and conductivity, among others 70. Research reports have described the design of a chemically cross-linked polyacrylamide hydrogel containing carboxyl-modified Au metal nanoparticle (AuC)-stabilized liposomes for pH-responsive local antibacterial delivery 71. This hydrogel effectively sustains the release of AuC liposomes for up to 7 days, and in the acidic environment associated with skin infections (pH 4.5), the released liposomes exhibit responsive fusion with bacterial membranes, achieving localized bactericidal effects. Highly ordered layer-by-layer metal nanoparticle-loaded hydrogels were synthesized through alternating deposition of gold nanoparticles and poly(diallyl dimethylammonium chloride) (PDDA), leading to enhanced drug release rates when subjected to a magnetic field stimulus within the range of 40 to 110 Hz 72. Natural hydrogels serve as ideal substitutes for the extracellular matrix (ECM) in tissue and cell engineering, yet their lack of conductivity limits their applications 22. Cellular activity and functionality are dependent on the conduction of intercellular electrical signals. Metal nanoparticles can provide the conductivity that hydrogels lack, significantly augmenting their ability to promote various cellular functions 73. For instance, the combination of gold nanorods with hydrogels effectively enhances cell adhesion and intercellular communication, elevating the volumetric conductivity and mechanical rigidity of the hydrogel scaffold. This leads to increased expression of cardiac cell phenotypes and maturity, holding significant implications for the construction of tissue-engineered skin and its application in wound treatment 73-75.

Furthermore, the environmental responsiveness of MNHH enables the monitoring of wound microenvironments and microorganisms. MNHH based on label-free surface-enhanced resonance Raman scattering (SERRS) can be utilized to monitor wound infections caused by *Pseudomonas aeruginosa* 41. By incorporating uniformly dispersed Au nanorods, Au nanospheres, and Au nanostars within the hydrogel, forming distinct sensitivities and spatial resolutions, pyocyanin can be quantified, and its spatial distribution within biofilms can be imaged using the SERRS composite. Additionally, reports have demonstrated that Au nanostars and Pt nanospheres can bestow electrochemiluminescent immunosensing capabilities upon hydrogels for detecting human chorionic gonadotropin (HCG) 76. This method functionalizes HCG antibodies onto complexes based on Pt nanocarriers and sandwich-captured hormones between antibodies functionalized on Au nanocarriers. Under voltage stimulation, electrochemiluminescent signals are generated. These complexes can effectively detect changes in the steady state of wound microenvironments, aiding in predicting and guiding disease treatments. Metal nanoparticle composite hydrogels have been designed to construct a series of systems for monitoring steady-state changes, including variations in temperature, pH, and hydrogen peroxide concentration 77-80. Changes in wound pH values are closely linked to infection, often presenting an acidic environment (reduced pH) during skin infections. Research has discovered that by incorporating conductive Au metal nanoparticles, precise pH determination of hydrogels composed of dimethyl aminoethyl methacrylate (DMAEMA) and 2-hydroxyethyl methacrylate (HEMA) was achieved 81. Natural DMAEMA/HEMA hydrogels exhibit strong pH-responsive characteristics upon swelling but lack electrical conductivity. The introduction of gold nanocarriers imparts a pH-related electrochemical response to the hydrogel: as the pH shifts from acidic to neutral, the hydrogel swells, increasing interparticle distance and reducing the composite's conductivity, thereby accurately identifying pH changes 81.

In conclusion, MNHH effectively enhances the hydrogel structure, elevating its mechanical and self-healing properties, thereby rendering the hydrogel more malleable and pliable. This expansion in the capabilities of hydrogel dressings in wound care is attributed to MNHH. Moreover, MNHH represents a modifiable and controllable drug release system, enabling precise and intelligent drug delivery. Importantly, metal nanoparticle carriers bestow novel functional attributes upon hydrogels. These composites not only respond to local environmental changes but also allow triggering through external stimuli such as light, heat, and magnetism. They enable the construction of multifunctional composite hydrogels equipped with automatic sensing, real-time monitoring, and controllable intervention. Therefore, MNHHs possessing these new attributes transcend being merely dressings for infection control and wound healing promotion. They have emerged as intelligent and adaptable interfaces for wound management, paving the way for precise and intelligent wound treatments.

### 3.2 Hydrogels enhance the functionality of metal nanoparticles

Metal nanoparticles have been widely employed in wound healing applications 33,85,86. However, their application still faces numerous drawbacks, such as inadequate biocompatibility, a tendency to aggregate over time, impacting their biological performance, susceptibility to protein fouling and subsequent macrophage clearance, and toxicity due to accumulation within tissues 15,16,87. Moreover, metal nanoparticles often fail to distinguish between different bacterial strains, potentially harming beneficial microorganisms essential for ecological balance. For example, studies by Hussain *et al*. revealed that even low-level exposure to Ag nanoparticles induced oxidative stress and mitochondrial dysfunction in rat liver cells 88. Another investigation demonstrated the toxic impact of Ag nanoparticles on cytokine proliferation and expression in peripheral blood mononuclear cells 89. In contrast, hydrogels provide a means to address these shortcomings of metal nanoparticles. Firstly, stimulus-responsive hydrogels can alleviate the potential toxicity associated with metal nanoparticles. When loaded within hydrogels, metal nanoparticles can release slowly, reducing tissue accumulation and subsequent toxicity. Research reports the synthesis of a pH-responsive Ag-nanoparticle dendritic polymer system that releases drugs in response to infection-induced pH changes, displaying excellent antibacterial activity alongside minimal cell toxicity 82. Further studies suggest that incorporating Ag nanoparticles into a multilayer hydrogel system with PEG and heparin reduces hemolytic activity and minimizes damage to normal tissues 83. Additionally, hydrogels can enhance the long-term stability of metal nanoparticles 90. Copper nanoparticles can generate antibacterial effects similar to those of Ag+ ions, but reduced oxidative activity compromises their stability and antibacterial efficacy. Incorporating Cu nanoparticles within a hydrogel matrix shields their surface from ROS impact while retaining antibacterial activity 84. Thus, metal nanoparticle hybrid hydrogels can effectively mitigate limitations associated with metal nanoparticles for wound healing applications, leveraging their advantages while promoting wound healing. However, it is important to note that despite the reduced tissue accumulation and toxicity facilitated by hydrogels, there remains a possibility of prolonged deposition and toxic effects in organs such as the skin, liver, and kidneys with prolonged usage 33,35. Therefore, further research into degradable metal materials is warranted to enhance the biocompatibility of metal nanoparticle-loaded hydrogels.

### 3.3 The synergy between hydrogels and metal nanoparticles

The combination of hydrogels and metal nanoparticles not only compensates for each other's deficiencies but also generates a powerful synergistic effect, such as synergistic antibacterial and wound healing properties.

**Figure 2:**
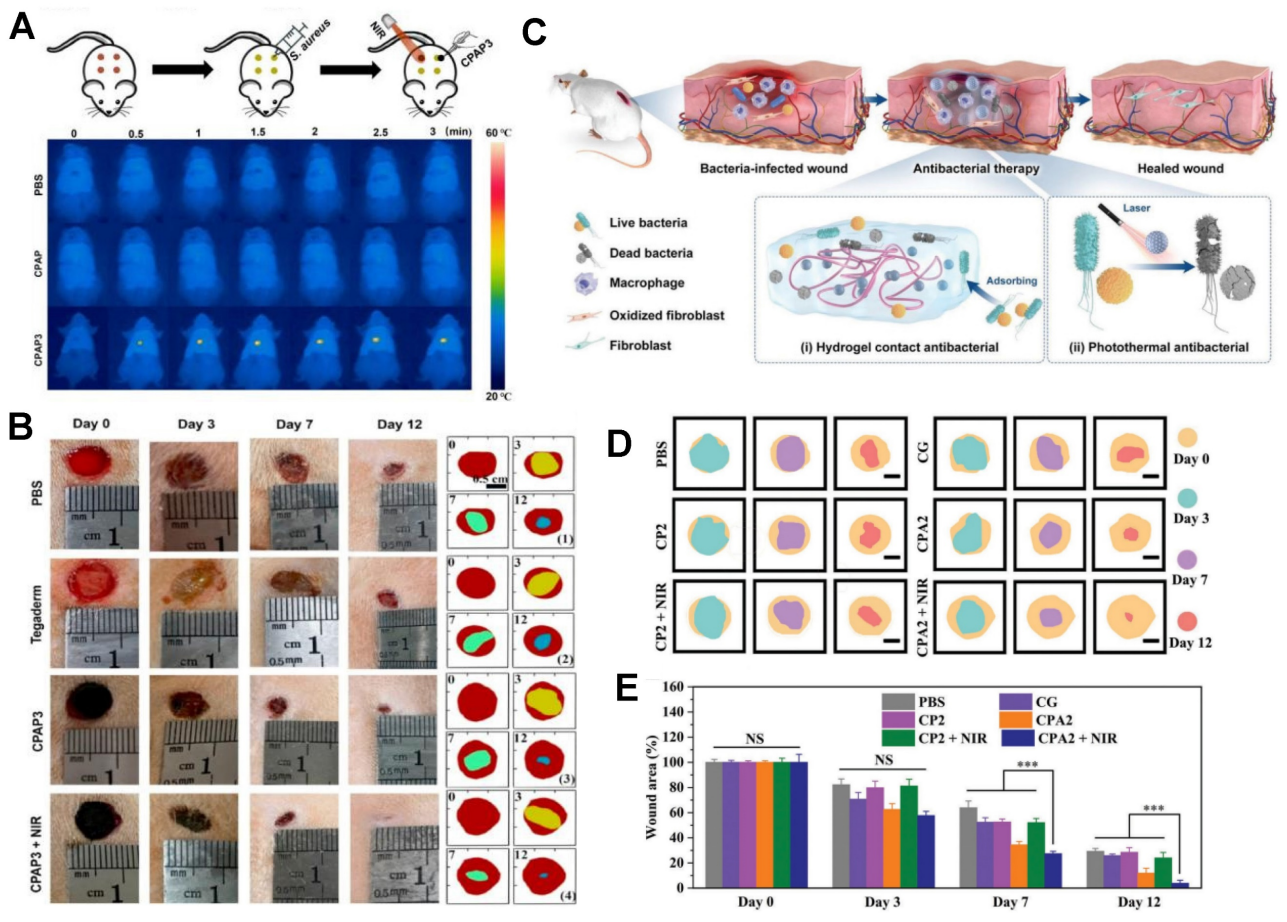
Metal nanoparticle hybrid hydrogels for treating infected wounds. (A)Schematic diagram of in vivo wound treatment evaluation of CPAP/PDA@Cu hydrogel photothermal performance, along with infrared thermographic images; (B) Photographic images and schematic illustration of wound contraction during the treatment of *Staphylococcus aureus*-infected wounds from Day 0 to Day 12: (1) PBS, (2) Tegaderm, (3) CPAP3 and (4) CPAP3 + NIR. Reproduced with permission from 94. Copyright 2022, Elsevier. (C) Schematic representation of the fabrication of the CG/PDA@Ag hydrogel and its application as a photothermal antibacterial platform for wound dressings. (D) Schematic diagram depicting wound contraction from Day 0 to Day 12 with different treatments for *Staphylococcus aureus*-infected wounds. (E) Wound area for each group (n = 3, ** p < 0.01, ***p < 0.001, NS indicates not significant). Reproduced with permission from 91. Copyright 2022, Wiley-VCH GmbH.

The collaboration between hydrogels and metal nanoparticles exhibits a robust synergistic antibacterial function, which is illustrated in Figure 2. Hydrogels efficiently capture bacteria and, when combined with metal nanoparticles, achieve precise pathogen eradication. Moreover, encapsulating metal nanoparticles within hydrogel matrices significantly enhances their photothermal capability, leading to synergistic antibacterial activity. For instance, by employing a reduction deposition method, Ag nanoparticles grown on the surface of polydopamine (PDA) nanoparticles displayed a remarkable increase in photothermal conversion efficiency to 36.1% (PDA @ Ag). Further embedding PDA@Ag nanoparticles into a polysaccharide (cationic guar gum, CG) network produced CG/PDA@Ag hydrogels 91. As shown in Figure 2, the PDA@Ag nanoparticles were uniformly dispersed without aggregation, and the photothermal conversion efficiency of the designed CG/PDA@Ag system increased from 36.1% to 38.2% 91. Additionally, CG polysaccharide hydrogels, rich in functional hydroxyl and quaternary ammonium groups, interact with bacteria through electrostatic forces, van der Waals forces, and hydrophobic interactions, leading to nonselective capture and elimination of both gram-positive and gram-negative bacteria 91. This provided supplementary antibacterial effects.

Furthermore, hydrogels and metal nanoparticles synergistically promote wound healing. Hydrogels effectively absorb wound exudate, prevent infection, and create a favorable microenvironment for healing 16,18-20. They support and accelerate cell migration, proliferation, and tissue repair 21-23. Metal nanoparticles, offering nanoenzyme activities similar to glucose oxidase and catalase, enhance the wound microenvironment. Their combined action significantly advances wound healing. For example, a combination of silver nanoparticles and epigallocatechin gallate synergistically regulated the expression of growth factors at injury sites, improving wound healing outcomes 92. Additionally, composite materials combining gold nanoparticles with natural hydrogels have been shown to promote fibroblast migration and granulation formation and reduce inflammatory factors 37,93. Studies have also demonstrated that an alloy of Au-Pt nanoparticles combined with oxidized hyaluronic acid (OHA) and carboxymethyl chitosan (CMCS) exhibited outstanding antibacterial and self-healing properties in hydrogel dressings (OHCs). The Au-Pt alloy nanoparticles acted as simulated glucose oxidase and catalase, enhancing wound microenvironment modulation 93. Composite hydrogel dressings significantly improved wound microenvironments and accelerated the healing of diabetic wounds. It is important to note that while metal nanoparticles often endow hydrogels with excellent photothermal capabilities, appropriate temperatures can effectively promote wound microcirculation, elevate local oxygen levels, and accelerate wound healing.

The combination of metal nanoparticle-hydrogel complexes shows robust synergistic antibacterial and wound healing capabilities. They release potent metal ions for antibacterial effects, establish temporary barriers against pathogen invasion, regulate cell proliferation and differentiation, and achieve precise pathogen eradication through photothermal effects. As a potential solution to antibiotic resistance, composite antibacterial hydrogels hold promising prospects in the field of wound healing.

## 4. Various types of MNHH

As shown in Table 2, various types of metal nanoparticles or nanomaterials containing metal components have been utilized in the preparation of MNHH. These include silver nanoparticles, gold nanoparticles, copper nanoparticles, and more. These hydrogels exhibit excellent mechanical properties, antimicrobial capabilities, and wound healing promotion, making them extensively applied in clinical wound management. The following sections will describe the characteristics and potential applications of different types of MNHH based on the types of metals used.

### 4.1 MNHH based on Monometallic Nanoparticles

#### 4.1.1 Silver nanoparticle hybrid hydrogel

Throughout history, silver has been recognized for its antibacterial properties. Silver nanoparticles (Ag NPs) exhibit potent antimicrobial activity against a wide range of microorganisms, including drug-resistant bacteria, fungi, and viruses. Ag NPs utilize various mechanisms for antibacterial action, although a definitive mechanism is still under debate. The integration of silver nanoparticle carriers with hydrogels effectively prevents pathogenic colonization at wound sites. While the antibacterial mechanisms of Ag NPs remain contentious, their robust broad-spectrum antibacterial efficacy is undeniable. Currently, the antibacterial mechanisms of Ag NPs can be categorized into six types: cell membrane disruption, inhibition of the bacterial respiratory chain, induction of bacterial genetic toxicity, interference with bacterial protein synthesis and folding, induction of oxidative stress and ROS production, and light-induced damage to bacterial proteins 33,95.

**Figure 3:**
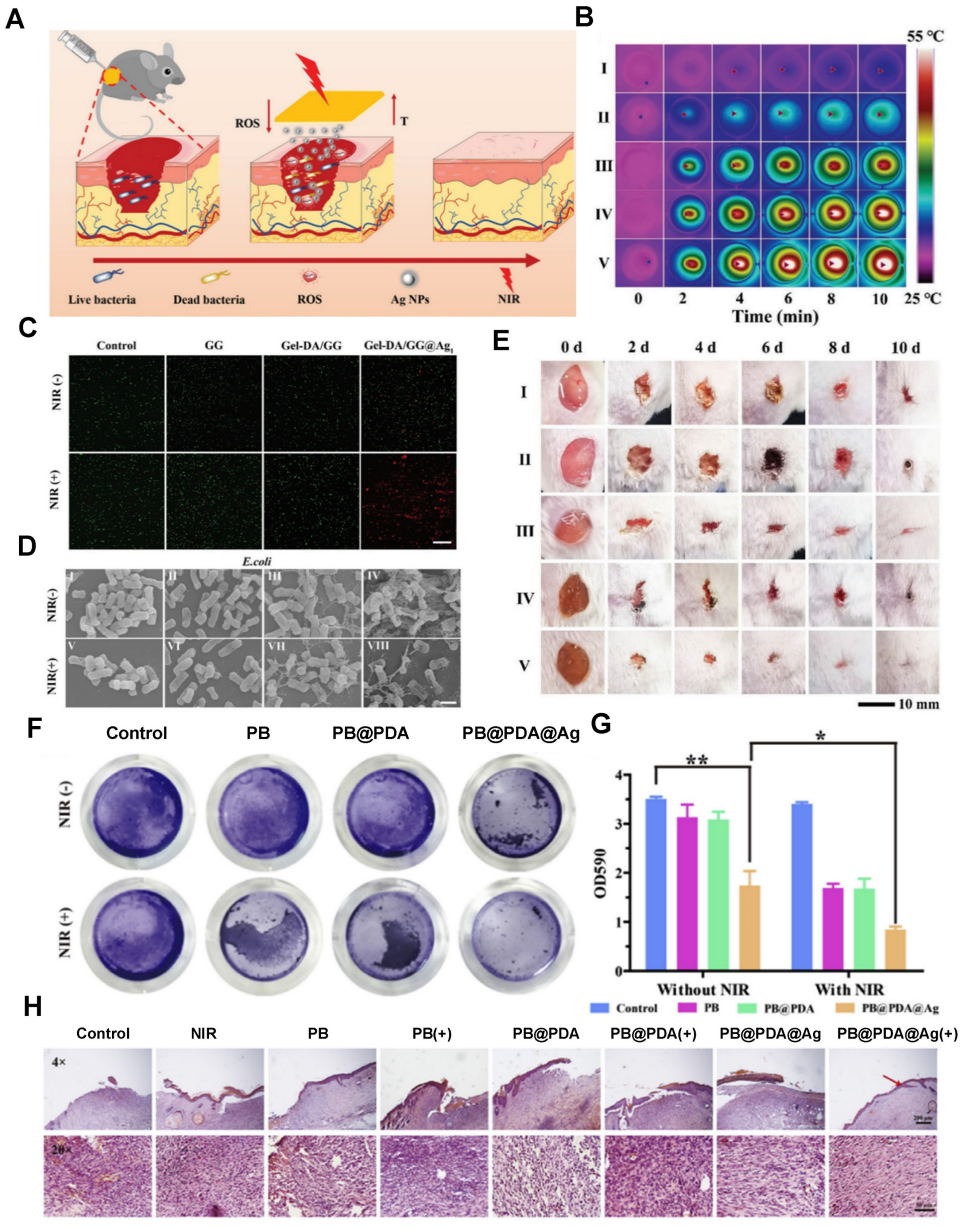
MNHH based on monometallic nanoparticles is used for wound treatment. (A) Application of the Gel-DA/GG@Ag hydrogel to wound healing. (B) Photothermal effects of the Gel-DA/GG@Ag hydrogels. (C) Fluorescence images of *S. aureus* treated with different hydrogels. (D) Morphologies of *E. coli* treated with I) Control, II) GG, III) Gel-DA/GG, IV) Gel-DA/GG@Ag1, V) control + NIR, VI) GG + NIR, VII) Gel-DA/GG + NIR, and VIII) Gel-DA/GG@Ag1 + NIR. Irradiation time: 10 min. Scale bars, 1 µm. (E) Photographs of *S. aureus*-infected wounds with different treatments. I) Control, II) GG, III) Gel-DA/GG, IV) Gel-DA/GG@Ag1, and V) Gel-DA/GG@Ag1 + NIR. Reproduced with permission from 116. Copyright 2021, Wiley‐VCH GmbH. (F) Photographs of crystal violet-stained MRSA biofilms treated with different samples, including normal saline, NIR, PB, PB + NIR, PB@PDA, PB@PDA + NIR, PB@PDA@Ag, and PB@PDA@Ag + NIR. (G) Relative MRSA biofilm biomass treated with different materials. MRSA biofilm biomass was determined by measuring the absorbance at 590 nm. (H)H&E staining images of infected wound tissue after various treatments for 8 days. Reproduced with permission from 97. Copyright 2020, Elsevier.

Research indicates that the primary mechanism of antibacterial action of Ag NPs (silver nanoparticles) is the release of silver ions (Ag^+^). Composite materials of silver nanoparticle carriers and hydrogels exhibit potent antibacterial effects, effectively inhibiting common wound pathogens such as *Escherichia coli* and *Pseudomonas aeruginosa* while promoting wound healing. For instance, Baukum and colleagues employed solvent casting to create SA/gelatin hydrogels with crosslinked silver nanoparticle carriers using varying concentrations of calcium chloride (CaCl_2_) 96. These doped Ag NPs significantly enhanced the mechanical properties of the hydrogel and endowed it with excellent antibacterial activity against *Staphylococcus aureus*, *Pseudomonas aeruginosa*, and *Escherichia coli* 96. As illustrated in Figure 3, a composite PB@PDA@Ag containing silver nanoparticle carriers was prepared to treat MRSA (methicillin-resistant *Staphylococcus aureus*) infections in a diabetic model with laser assistance 97. This combination strategy demonstrated synergistic effects in combating MRSA through cell membrane disruption, ROS production, ATP reduction, and oxidative GSH reduction, effectively accelerating wound healing in diabetic MRSA-infected wounds. Silver nanoparticle-hybrid hydrogels can establish physical barriers, prevent microbial invasion, reduce infection risks, and support fibroblast migration, thereby accelerating wound healing. For instance, Masood *et al*. reported the preparation of an Ag nanoparticle carrier-chitosan-polyethylene glycol (PEG) composite hydrogel. Compared to pure hydrogels, this Ag nanoparticle carrier hydrogel exhibited improved mechanical strength and physical properties. It better maintained a microenvironment conducive to wound healing, accelerating wound healing in diabetes-induced rabbit models 98. Moreover, hydrogels can enhance the stability of silver nanoparticles, thus prolonging their antimicrobial activity. For example, Obradovic *et al*. optimized a freeze‒thaw technique based on alginate, polyvinyl alcohol (PVA), and poly(N-vinylpyrrolidone) (PVP) to produce Ag nanoparticle-loaded composite alginate microsphere hydrogels. This approach suppressed the tendency of silver nanoparticle carriers to aggregate, ensuring their stability 99. Furthermore, silver nanoparticle-hybrid hydrogels exhibit superior mechanical properties and physicochemical characteristics compared to natural hydrogels 100.

Silver nanoparticle hybrid hydrogels possess numerous advantages, including antimicrobial properties, tissue regeneration promotion, and excellent mechanical performance, making them highly promising multifunctional wound dressings for enhancing wound healing. However, the clinical application of silver nanoparticle-hybrid hydrogels still faces challenges. First, the inhibition of gram-positive bacteria by silver nanoparticle carriers is inferior to their effect on gram-negative bacteria, possibly due to the stronger resistance of gram-positive bacteria's peptidoglycan cell walls. Second, silver nanoparticle carriers are cytotoxic to normal cells as well, and their accumulation in natural environments or human tissues such as skin, liver, and kidneys can pose potential toxicity risks in the long term. Last, despite the rarity of bacterial resistance to silver ions, with the increasing clinical use of silver nanoparticle-hybrid hydrogel materials, this issue remains worthy of attention. In designing silver nanoparticle carrier hydrogel composites, several aspects should be considered: enhancing the antibacterial capacity against gram-positive bacteria, prolonging the release time of antibacterial components, minimizing cytotoxicity to cells, and developing environmentally friendly in situ synthesis processes to avoid potential environmental and health concerns 7,32,90,101.

#### 4.1.2 Gold nanoparticle hybrid hydrogel

Although gold is generally considered biologically inert, gold nanoparticle carriers exhibit various biological functions and have been widely applied in the medical field. Compared to other metal nanoparticle carriers, such as silver and copper, gold nanoparticle carriers have minimal toxicity to human tissues and cells 71. Gold nanoparticle carriers can be designed in different sizes or shapes, and when combined with hydrogels, they create biomaterials with enhanced mechanical properties, environmental responsiveness, biocompatibility, conductivity, and antimicrobial performance for wound management 102-106.

Most hydrogel scaffolds themselves are nonconductive, requiring additives to facilitate intercellular signal transmission, thus limiting their applications in the field of biotechnology 107. Metal nanocarriers offer hydrogel conductivity enhancement while concurrently augmenting their mechanical performance, significantly advancing the applicability of hydrogel scaffolds in tissue engineering. Research has revealed that embedding gold nanocarriers within chitosan hydrogels generates scaffolds with electrical conductivity akin to cardiac tissue 108,109. Furthermore, the concentration of gold nanocarriers directly impacts scaffold conductivity and mechanical rigidity, thereby augmenting the phenotype expression and maturity of cardiac cells 108,109. This composite material has been shown to enhance the regenerative capacity of myocardial cells post myocardial infarction, a finding of paramount significance for constructing engineered skin tissues in vitro 108,109. Beyond the fortification of mechanical and conductive attributes, gold nanocarriers can also promote wound healing by inhibiting the growth of bacteria and fungi or stimulating the release of wound healing factors. Research has demonstrated that the antibacterial mechanism of gold nanocarriers against *Escherichia coli* involves hindering ATPase activity to disrupt membrane potential, concurrently suppressing the connection between ribosomal subunits and tRNA 110. Gold nanocarriers can attach to bacterial membranes, leading to leakage of bacterial contents or permeation through the outer membrane and peptidoglycan layer, resulting in bacterial demise. Zahra and colleagues reported a composite of gold nanocarrier hydrogel synthesized through sunlight-assisted green methods, which exhibited heightened antibacterial activity. Moreover, this composite elevated the levels of the transcription factor NANOG and transmembrane adhesive protein CD34 in mouse wound sites, consequently promoting wound healing 55. Furthermore, research has unveiled noticeable variations in antibacterial activity between gold nanocarriers of distinct shapes. Gold nanostars and gold nanoflowers exhibited greater antibacterial activity than gold nanospheres, with rod-shaped nanocarriers more effectively disrupting pathogenic biofilms. This observation may be attributed to the distinctive shapes of gold nanocarriers providing higher surface area-to-mass ratios and facilitating interaction with cell membranes 111,112.

Apart from their inherent antibacterial capabilities, gold nanogels can also be utilized for photothermal therapy (PTT) to combat bacterial infections. PTT, an effective strategy for treating bacterial infections using photothermal materials under visible light or near-infrared radiation, leverages tunable surface plasmon resonance effects under near-infrared radiation. Gold can be shaped into specific configurations and widely employed for PTT therapy 113. Research has developed a novel soy protein-based hydrogel by utilizing oxidized dextran as a crosslinker and loading it with gold nanocarriers 114. This hydrogel exhibited robust mechanical performance and photothermal antibacterial abilities against *Escherichia coli* and *Staphylococcus aureu* 114. While photothermal therapy effectively counters bacterial infections, overheating may inadvertently damage nearby host cells or tissues. Therefore, Chen and collaborators devised a localized precise photothermal antibacterial strategy termed Thermal Destruction-Induced Relief of Interface 115. This strategy involves a flexible base of polydimethylsiloxane with microchannels and a temperature-sensitive PNIPAm hydrogel loaded with gold nanocarriers 115. The PNIPAm hydrogel undergoes contraction under near-infrared radiation, confining the heat generated by gold within the microchannels, thus achieving precise therapeutic aims 115.

Although the current antibacterial efficacy of gold nanocarriers may not match that of silver nanocarriers, gold nanocarriers possess unique advantages, bestowing upon gold nanogels a broad spectrum of antibacterial properties, low cytotoxicity, superior conductivity, and mechanical strength. As a potentially influential adjunct, gold nanogel scaffolds have the potential to facilitate wound healing intelligently and precisely. When designing composite materials involving silver nanocarrier hydrogels, it is imperative to consider the following: enhancing antibacterial efficacy against gram-positive bacteria, prolonging the release duration of antibacterial agents, minimizing cytotoxicity, and developing environmentally and health-friendly in situ synthesis methods, as suggested by the literature 7,32,90,101.

### 4.2 MNHH based on metal oxide nanoparticles

**Figure 4:**
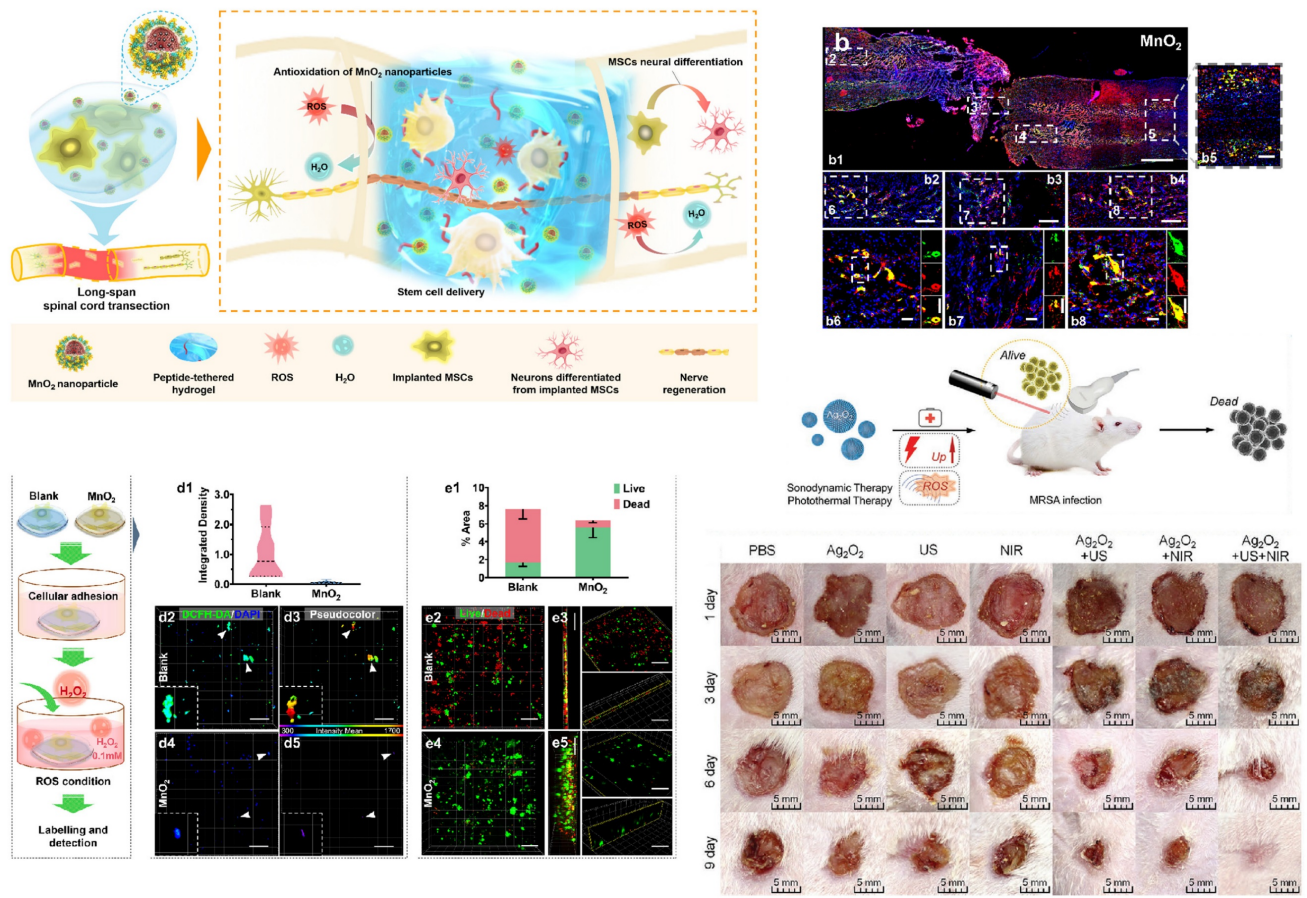
Applications of metal oxide nanoparticles. (A) MnO_2_ NPs improve the survival, integration and neural differentiation of transplanted MSCs and promote nerve tissue regeneration by mitigating the oxidant microenvironment. (B) Nerve fibers were significantly regenerated by the MnO_2_ nanoparticle (NP)-dotted hydrogel across the whole tissue (b1) with integrated MSCs (green) colocalizing with NF (red), the marker of mature neurons. MSCs integrating into the distant (b2) and adjacent (b4) regions exhibited a larger total amount of 21 wells and a higher proportion of neural differentiation compared to those sustained in the lesion site (b3). Caudal distant segments of the tissue (b5) showed the same result as that in the rostral side (b2). Micrographs in (b6-b8) show magnified views of the boxed fields in (b2-b4). Separated NF and GFP-MSC channels of the boxed cells in (b6-b8) are shown in detail and presented on the right of each micrograph. Scale bar, 1 mm (b1), 200 μm (b2-b5), 50 μm (b6-b8). (C). MSCs were encapsulated in the hydrogels and allowed to adhere overnight before being exposed to the ROS microenvironment-simulating medium containing 0.1 mM H_2_O_2_. (D) After 24 h, the intracellular ROS levels of MSCs were quantified (d1) in the blank (d2, d3) and MnO_2_ NP-dotted (d4, d5) hydrogels after dichlorodihydrofluorescein diacetate (DCFH-DA) labeling (n=3), demonstrating the effective antioxidant impact of the MnO_2_ NP-dotted hydrogel on MSCs. Green, DCFH-DA; blue, DAPI. The fluorescence of DCFH-DA in (d2, d4) was analyzed using pseudocolor (d3, d5) according to mean intensities to further show more detailed differences. € Cytoviability of the MSCs after 24 h of incubation was analyzed by Live/Dead assay. Numbers of live cells (green) and dead cells (red) were quantified in terms of stained areas (e1). Data in the graph are presented as the average ± SD (n= 9 views). Representative images of the cells in the blank (e2, e3) and MnO_2_ NP-dotted (e4, e5) hydrogels are presented, with n= 3 hydrogels for each group. Scale bar, 100 μm (D and E). Reproduced with permission from 136. Copyright 2019, American Chemical Society. (F) Schematic illustration of the combined antibacterial sonodynamic and photothermal therapy in an MRSA-infected murine model with Ag_2_O_2_ NPs. (G) Representative images of wounds infected with MRSA in seven different treatment groups on days 1, 3, 6, and 9. Reproduced with permission from 137. Copyright 2021, Wiley‐VCH GmbH.

Metal oxides refer to binary compounds composed of oxygen and another metallic chemical element, encompassing alkaline oxides, acidic oxides, amphoteric oxides, and more. Compared to elemental metals, metal oxides often possess greater stability, and when nanosized, they exhibit various catalytic activities (Figure 4).

Beyond inert metals such as silver and gold, other metal nanoparticles can also combine with hydrogels in the form of oxides. These nanoparticles release metal ions continuously on wounds, achieving antimicrobial and wound healing effects. These metal compounds include ZnO, CuO, iron oxides, MnO_2_, and more 86,117,118.

Zinc, as the most commonly used antibacterial agent, employs several mechanisms against microorganisms. ZnO nanoparticles exhibit antibacterial activity at suitable concentrations without cytotoxicity, making them widely used in cosmetic production. ZnO hydrogels demonstrate great potential in drug delivery and wound healing promotion. CMC-Zn-MEL composites containing ZnO nanoparticles exhibit antibacterial activity against both gram-positive and gram-negative bacteria, making them excellent wound dressing materials 119. Despite relatively weaker antibacterial efficacy, ZnO nanoparticles possess low cytotoxicity, suggesting their potential for broader clinical applications. Moreover, research indicates that ZnO nanoparticles can facilitate bone tissue regeneration, implying their potential for tissue engineering applications 120,121.

Copper nanoparticles exhibit a bacterial killing efficiency comparable to that of silver nanoparticles. Although the antibacterial effect of hydrogels containing copper nanoparticles (Cu-nano/CuO-nano) is weaker than that of Ag-nano, they show broader spectrum antibacterial activity against fungi and bacteria 122-124. Recent studies on CMC/CuO-nano composite hydrogels and CS hydrogels loaded with copper particles have demonstrated excellent antibacterial effects against *E. coli* and *S. aureus* without inducing toxicity 125,126. In wound environments, copper nanoparticles are prone to oxidation, which reduces their stability and antibacterial efficacy. Incorporating copper nanoparticles into hydrogels can protect them from ROS effects, maintaining their antibacterial activity 127,128. In addition to releasing copper ions for bactericidal effects, copper nanoparticle hydrogels can utilize the local surface plasmon resonance (LSPR) effect for photothermal pathogen elimination. Researchers have developed Cu nanoparticles using a polyol method and then incorporated them into biocompatible polysaccharide hydrogels, enhancing the stability of Cu nanoparticles. These hybrid hydrogels exhibit rapid self-healing capabilities and exceptional photothermal antibacterial performance 129. Other studies have reported the in-situ formation of copper nanoparticles on polydopamine surfaces, followed by introduction into precursor polyelectrolyte hydrogels (CPAPs) to prepare hybrid hydrogels CPAP/PDA@Cu. This composite material prevents nanoparticle aggregation, demonstrating excellent photothermal antibacterial properties (PCE 55.4%) and electrostatic adsorption capacity for bacterial capture 94. Such adaptive and potent antibacterial dressings hold significant potential for treating infected wounds 94.

Other metal oxide hydrogel composites also exhibit various biological functions, including antibacterial properties, stimuli-responsive drug release, and tissue regeneration promotion. Recent reports have indicated that hydrogels containing magnesium oxide nanoparticles also combat microorganisms through multiple mechanisms 130-132. Hesaveh *et al*. incorporated MgO nanoparticles into hydrogels derived from hydroxypropyl κ-carrageenan to control drug delivery in gastrointestinal studies 133. Furthermore, research has employed chitosan hydrogel microbeads containing superparamagnetic iron oxide nanoparticles (SPIONs) for stimulus-responsive release of vancomycin 134. Metal oxides can also confer characteristics such as magnetic heating to hydrogels, further expanding the application scope in wound management, artificial skin, and more. Research has embedded GO-Fe3O4 nanoparticles in poly(N-isopropylacrylamide), alginate-interpenetrated polymer hydrogels, developing an innovative nanocomposite hydrogel microcapsule drug delivery system 135.

These metal nanoparticle hybrid hydrogels (MNHHs) offer multiple effective pathways to combat infections, with few reported cases of bacterial resistance. They prove to be efficient strategies against antibiotic-resistant microorganisms. The stability of metal nanoparticles ensures sustained antimicrobial effects, even after release from dead cells, allowing them to continue eradicating other microbial cells. Furthermore, composite materials can achieve pathogen eradication through photothermal effects and offer controlled drug delivery systems locally through stimulus-responsive behaviors. Last, such composite materials exhibit superior biocompatibility, enhanced mechanical strength, flexibility, and self-healing properties, making them ideal candidates for creating wound coverings.

### 4.3 MNHH based on multiple metal materials

In addition to loading single-metal nanoparticle hydrogels, there is also research focused on creating composite hydrogels containing two or more types of metal nanoparticles. Compared to hydrogels with single-metal nanoparticles, the synergistic effects arising from alloy interactions introduce many pleasantly surprising new properties to these composite materials: enhanced antimicrobial efficiency, reduced cytotoxicity, and heightened stimulus-responsive capabilities 85,138-140.

Multimetal nanoparticle composites can be utilized to enhance antimicrobial efficiency while simultaneously reducing their toxicity to cells. Research has been conducted using gold-silver nanoparticles with an average size of 10 nm loaded into chitosan to create CS-Au-Ag wound dressings 85. CS-Au-Ag wound dressings exhibited faster, higher, and more sustained release of silver ions compared to chitosan dressings loaded with the same silver content (CS-Ag), thus demonstrating enhanced antimicrobial activity 85. These findings suggest that reducing the silver content can mitigate the cell toxicity that limits further applications of silver nanoparticles, making them a promising material for wound dressings 85. Multimetal composites have also exhibited good antimicrobial activity against multidrug-resistant bacteria 141. Research has employed biopolymer starch as a reducing and capping agent for the environmentally friendly, repeatable, and simple synthesis of Ag/Au bimetallic nanoparticles. These nanoparticles demonstrated excellent antimicrobial performance against multidrug-resistant *Escherichia coli* and methicillin-resistant *Staphylococcus aureus*, promoting wound healing and reducing scar formation 142. Furthermore, Imran *et al*. utilized zinc oxide (ZnO) coverage on biogenic gold nanoparticles obtained from Hibiscus plant extracts to synthesize Au@ZnO core-shell nanocomposites 140. The synthesized nanocomposites aided in generating reactive oxygen species (ROS), exhibiting antibacterial and antibiofilm activities against *Staphylococcus aureus* and methicillin-resistant *Staphylococcus aureus* (MRSA) 140. Simultaneously, these nanocomposites reduced their toxicity toward mouse fibroblasts under normal and high glucose conditions, promoting wound healing 140. Similarly, research designed Ag/CaO nanocomposites (NCs) that demonstrated effective antibacterial activity against *Staphylococcus aureus* and MRSA 143. The formation of NCs maintained the antibacterial efficacy of silver nanoparticles while decreasing their toxicity to mammalian cells 143.

Alloy interactions can also enhance metal-stimulus responsiveness. It has been found that combining silver nanoparticles with iron oxide confers new magnetic responsiveness to the material 144. The presence of iron imparts magnetic responsiveness to Ag/Fe_3_O_4_ NCs, allowing them to penetrate and disrupt biofilms under a magnetic field, effectively inhibiting wound infections 144. Compared to pure silver nanoparticles, Ag/Fe_3_O_4_ NCs exhibit lower silver release, less ROS generation, lower cell toxicity, and higher antibacterial efficacy 144. This presents a potential avenue for developing novel treatment strategies for chronic wound infections. Additionally, studies have reported combining silver with nanomaterials such as ZnO and MgO, resulting in superior photocatalytic and antibacterial activities 145-147.

Therefore, multimetal nanoparticle hybrid hydrogels can introduce new environmental responsiveness for precise therapeutic applications. Through their synergistic effects, diverse physicochemical properties, and varied mechanisms, bimetallic nanocomposite hydrogels, synthesized by combining two distinct metals, showcase enhanced antibacterial efficiency and lower tissue toxicity compared to monometallic hydrogels. They have emerged as effective tools to combat emerging drug-resistant bacteria and promote wound healing.

## 5. The application of metal nanoparticle-hybrid hydrogels in wound healing

An ideal wound dressing aims to fulfill the following functions: maintaining moisture, allowing gas exchange, preventing microbial growth, providing insulation, ensuring biocompatibility, offering mechanical strength, ensuring sterility, being easy to remove, and cost-effectiveness. Apart from these fundamental considerations, modern dressings should also possess attributes such as absorbing exudate, aiding cell adhesion and proliferation, and loading and releasing antimicrobial agents or growth factors as therapeutic agents. Loaded metal nanoparticle hydrogel materials exhibit excellent mechanical properties, potent antimicrobial capabilities, environmental responsiveness, the ability to regulate cell differentiation and proliferation, and remarkable tissue compatibility. These characteristics make them ideal materials for creating antimicrobial wound dressings and tissue-engineered skin. As depicted in Table 3, these composite materials have been widely employed in the treatment and management of chronic wounds and burn injuries.

### 5.1 Chronic wounds

Chronic wounds, such as diabetic foot ulcers and pressure sores, often result in complications due to slow healing and an increased risk of infection. In such cases, microbial invasion further delays healing, creating a vicious cycle. Bacteria that colonize within the wound can rapidly develop into biofilms. The presence of biofilms varies significantly on the foundation of skin damage. These biofilms hinder wound healing through pathways such as inhibiting epithelialization, inducing prolonged chronic inflammation in the local environment, promoting cell apoptosis, and generating reactive oxygen species (ROS) 188-190. Therefore, treating chronic wounds necessitates not only promoting recovery but also prioritizing pathogen inhibition. Wound dressings should possess enhanced antibacterial properties to address these challenges 5.

**Figure 5:**
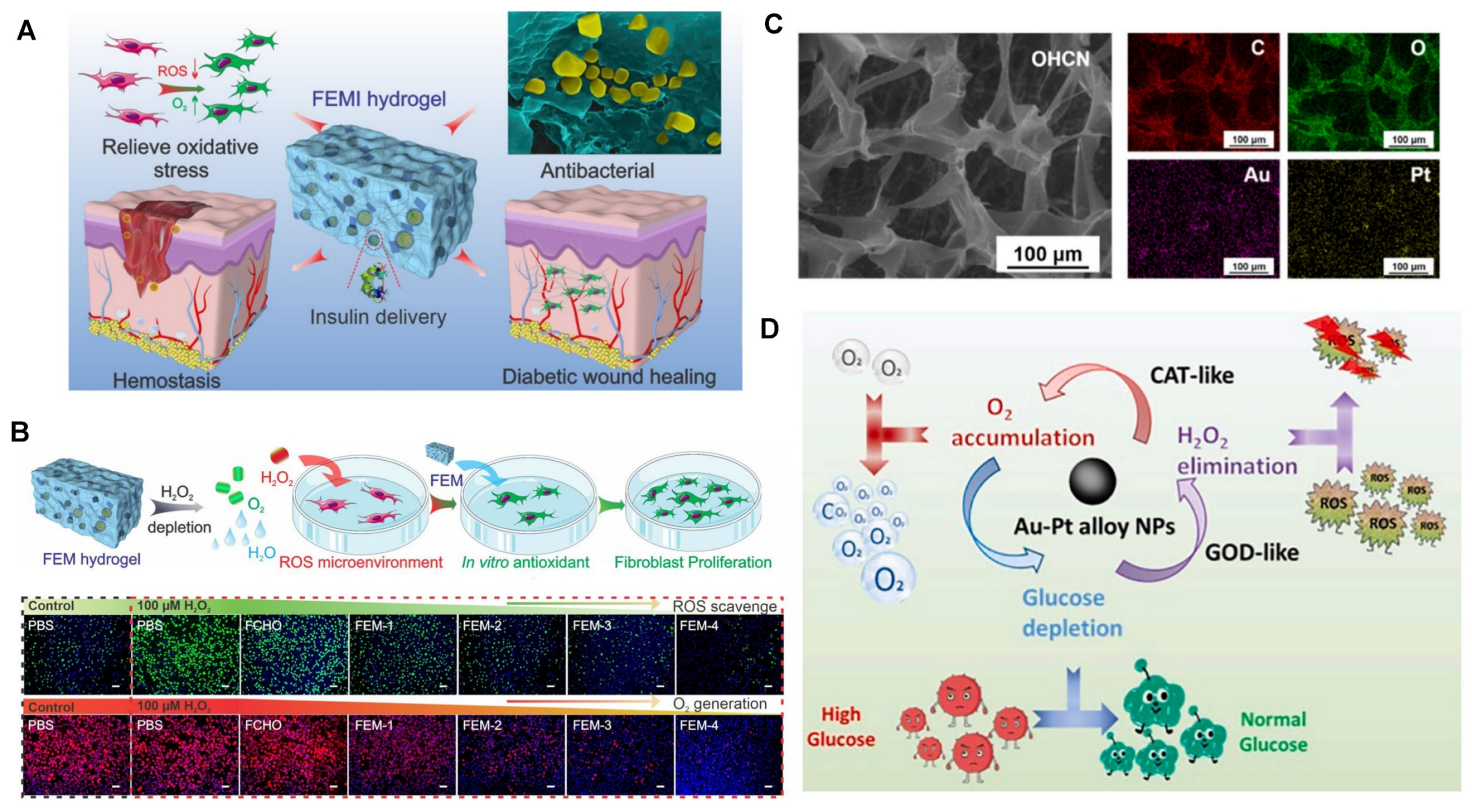
The application of metal nanoparticles hybrid hydrogels in the treatment of chronic wounds. (A) The FEMI hydrogel constructed an instructive microenvironment to ameliorate inflammation, accelerate cell proliferation, promote angiogenesis, enhance granulation tissue formation and re-epithelialization, and stimulate MDR bacteria-infected diabetic wound healing in vivo. (B) Oxygen generation catalyzed by the MnO2 nanosheets in the FEM hydrogel alleviated oxidative stress in L929 cells. Reproduced with permission from 162. Copyright 2020, American Chemical Society. (C) Representative SEM and EDS mapping images of the Au-Pt alloy NPs@OHC hydrogel. (D) The mutually reinforcing mechanism of GOD-like and CAT-like activities of Au-Pt alloy NPs, through which they effectively enhanced glucose depletion, ROS elimination and O2 generation. Reproduced with permission from 138. Copyright 2022, Elsevier.

Metal nanoparticle hybrid hydrogels, with silver nanoparticles (Ag NPs) as a representative, perfectly align with the therapeutic demands of chronic wounds by effectively restraining biofilm growth and creating a microenvironment conducive to wound healing (Figure 5). Silver nanoparticle hydrogels exhibit notable antibacterial effects against common wound pathogens such as *Staphylococcus aureus* and *Pseudomonas aeruginosa*, especially showing remarkable antibacterial efficacy against various drug-resistant strains 33. For instance, Reena *et al*. developed a quercetin (QCT)-Ag NP composite hydrogel for the synergistic treatment of chronic diabetic wounds 178. Compared to plain hydrogels, the QCT-Ag NP hydrogel exhibited enhanced therapeutic effects against *Staphylococcus aureus* and *Escherichia coli*, significantly accelerating wound healing in diabetic wound models and improving re-epithelialization 178. Apart from silver nanoparticle composites, gold nanoparticle hydrogel composites have also been shown to accelerate wound healing in diabetic wounds. Multiple studies highlight that composites combining gold nanoparticles with naturally derived materials such as cellulose, chitosan, alginate, and dextran can stimulate fibroblast migration and granulation while reducing wound inflammation 35,55,93,111. A multifunctional self-healing hydrogel dressing containing Au-Pt alloy nanoparticles was reported to regulate the complex wound microenvironment, accelerating diabetic wound repair 93. This material demonstrated excellent antibacterial and self-healing properties, along with functions such as blood glucose reduction and oxidative damage mitigation, significantly enhancing the pathological microenvironment and expediting the healing process of diabetic wounds 93. Therefore, metal nanoparticle hybrid hydrogels are effective in maintaining a moist, sterile wound environment, suppressing drug-resistant bacterial colonization, modulating cell proliferation and differentiation, and promoting wound healing. These hybrid hydrogels offer a promising solution for the treatment of chronic wounds such as diabetic foot ulcers.

### 5.2 Burns

The causes of death from extensive burns are often the result of bacteria infiltrating the body through the wound, leading to sepsis 191. Hydrogels are potent candidate materials for burn wound dressings due to their natural biocompatibility and biodegradability, promoting collagen deposition and wound contraction to aid healing. However, many hydrogels lack the mechanical strength and antibacterial efficiency required to create a sterile environment conducive to wound healing 15,16. Silver sulfadiazine is a standard antibacterial agent for burn wounds, but its topical use may cause various side effects, such as leukopenia, silver dermatitis, and kidney and liver toxicity, making it unsuitable for prolonged use 33.

Compared to traditional treatments, silver nanoparticle hydrogels demonstrate enhanced mechanical properties and excellent antibacterial and healing performances while avoiding the multiple side effects associated with conventional silver sulfadiazine. Silver nanoparticle hydrogels exhibit better antibacterial effects against multidrug-resistant bacteria. Research has incorporated Ag nanoparticles into thermosensitive methylcellulose (MC) hydrogels for treating burn wounds 182. In comparison to silver sulfadiazine cream, the composite inhibits bacterial cell proliferation, disrupts biofilm matrix stability, and displays stronger antibacterial efficacy with lower toxicity, thereby promoting burn wound healing 182. Further studies have developed a gel based on gelatin (GT) and silver nanoparticles (Ag NPs), which showcases high water absorption, along with effective antibacterial and antibiofilm activities to promote burn wound healing 180. The composite swelling rate is as high as 4000%, effectively absorbing wound exudates and allowing gas exchange. It exhibits excellent antimicrobial effects against methicillin-resistant *Staphylococcus aureus* (MRSA) and *Pseudomonas aeruginosa* (PA), common pathogens in burn wounds, effectively removing mature biofilms. This gel demonstrates superior hemostatic ability compared to commercial gelatin sponges and alleviates pain when dressings are removed from the wound 180.

Beyond infection control and promoting wound healing, composite materials can also provide real-time monitoring of burn wound infections through their environmental responsiveness. Recent studies have built gold nanoparticle hybrid hydrogels based on label-free surface-enhanced resonance Raman scattering (SERRS) for detecting infections by *Pseudomonas aeruginosa* in burn wounds 192. By uniformly embedding Au nanorods, Au nanospheres, and Au nanorods in a superlattice within the hydrogel, the composite creates various sensitivities and spatial resolutions to quantify pyocyanin, allowing for imaging of its spatial distribution within the biofilm 41. This holds significance for monitoring burn wound infections in patients and providing timely treatment.

Metal nanoparticle hybrid hydrogels combine the benefits of hydrogels and metal nanoparticles, demonstrating superior antibacterial effects and low cytotoxicity compared to traditional wound antibacterial agents. Moreover, these composites possess various environmental responsiveness traits, holding potential for creating multifunctional burn wound dressings with wound monitoring, intelligent drug release, and composite antibacterial properties.

### 5.3 Implants

Patients with extensive burns or chronic wounds often require long-term placement of catheters for corresponding treatments, and catheter-related infections are a common cause of sepsis in these patients 191.

Silver coatings effectively reduce the incidence of catheter-related infections, but silver-loaded and silver nanoparticle systems often exhibit hemolytic properties that hinder their long-term use 193. Silver nanoparticle hydrogels can mitigate hemolysis and enhance the biocompatibility of catheter coatings, maintaining the antimicrobial activity of silver nanoparticles while partially mitigating foreign body reactions. Studies have shown that incorporating Ag nanoparticles into a multilayer hydrogel system composed of PEG and heparin reduces its hemolytic activity 185.

Stimuli-responsive hydrogels provide another potential approach to mitigate silver toxicity. Research has designed a pH-responsive nanocomposite hydrogel by connecting oxidized polysaccharides with silver nanoparticles encapsulated in cationic dendritic polymers, which serve as a coating for implantable medical devices and exhibit antibacterial activity against both gram-positive and gram-negative bacteria 183. This composite can respond to pH reduction induced by bacterial infection and release antibacterial agents, displaying excellent antibacterial activity with minimal cytotoxicity 183.

The implantation of biomedical materials not only presents challenges in maintaining a sterile environment but also triggers the formation of collagen capsules through foreign body reactions. These reactions lead to permanent isolation of implants from surrounding tissues, hindering functionality. Research has found that PHEMA (poly(2-hydroxyethyl methacrylate)) and Ag-nanoparticle hydrogel implants exhibit good antibacterial performance against both gram-positive (*Staphylococcus aureus*) and gram-negative (*Escherichia coli*) bacteria, and by reducing immune reactions, they exhibit in vivo resistance to FBRs, completely preventing the formation of collagen capsules. This makes them an ideal material for next-generation implantable biomedical devices and tissue engineering scaffolds 184.

Metal nanoparticle hydrogels offer good biocompatibility, strong antimicrobial efficiency, and low cytotoxicity, making them ideal materials for producing medical implants such as catheters. They can effectively reduce catheter-related infections and have the potential to decrease the incidence of sepsis and mortality in patients with chronic wounds and extensive burns during perioperative periods.

### 5.4 Tissue engineering

While a range of mature tissue-engineered skin products have been utilized in clinical settings and various types of artificial skin have been developed, most of these products only exhibit structural similarity to human skin and possess the skin's barrier function. Due to the absence of skin appendages, they do not fully replicate the complete functions of natural skin, thus falling short of achieving true skin reconstruction. For instance, commonly used dermal substitutes in clinical applications often offer only limited coverage and are associated with high costs. MNHHs, with properties such as multifunctionality, environmental responsiveness, and antimicrobial properties, may present new ideas and directions in this area 74,194,195.

Natural hydrogels made from materials such as gelatin, collagen, and chitosan closely resemble the extracellular matrix (ECM) and serve as scaffolds in tissue engineering, playing various roles 22,74,194,195. However, their limited mechanical strength and electrical properties have restricted their applications 196. Cellular vitality in multicellular systems relies on close intercellular electrochemical signaling. The addition of metal nanoparticles enhances the mechanical and electrical properties of hydrogels, making these composite hydrogels ideal for tissue engineering.

**Figure 6:**
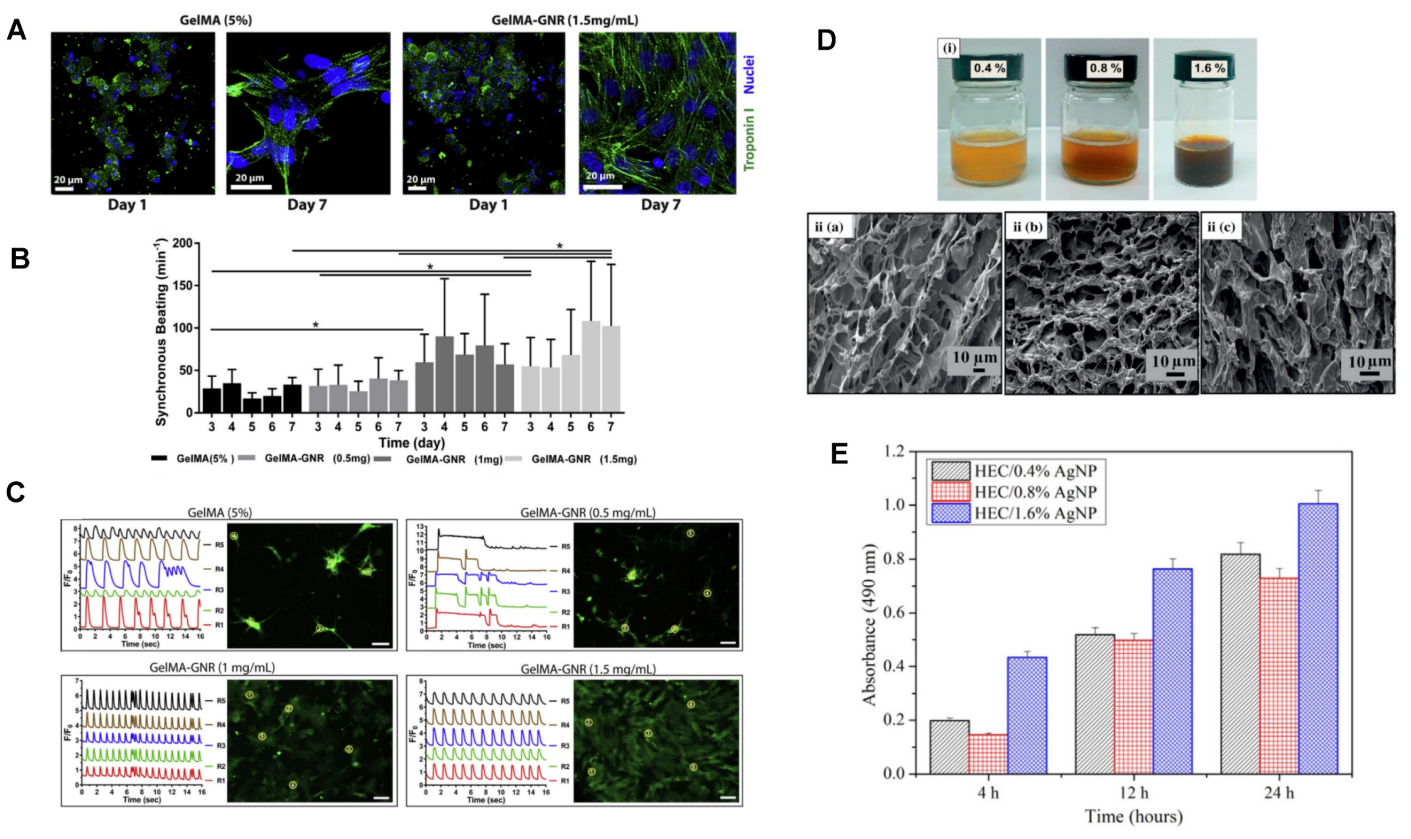
The application of metal nanoparticle hybrid hydrogels in tissue engineering. (A) Immunostained images of troponin I (green) on days 1 and 7 of culture for GelMA and 1.5 mg/mL GelMA-GNR hybrid hydrogels. (B) Synchronous beating frequency (beats per minute (BPM)) of cardiomyocytes from day 3 to day 7 of culture depicting robust and stable beating behavior in GelMA-GNR hybrids. A significantly higher number of beating frequencies was observed between highly GNR-concentrated hybrids compared to 0.5 mg/mL GelMA-GNR and pure GelMA hydrogels (*p < 0.05). (C) Calcium transient and extracted related frequency signals of intracellular changes in the concentration of Ca^2+^ within cultured cardiomyocytes for pure GelMA (5%), GelMA-GNRs (0.5 mg/mL), GelMA-GNRs (1 mg/mL), and GelMA-GNRs (1.5 mg/mL). R1 to R5 represent regions 1-5, and scale bars depict 100 μm. Reproduced with permission from 58. Copyright 2016 , Elsevier. (D) (i) Photograph of the HEC/Ag nanoparticle solutions obtained at different concentrations of AgNO3 and (ii) SEM micrographs of the HEC/Ag nanoparticle scaffolds at different concentrations of AgNO3 (a) 0.4%, (b) 0.8%, (c) 1.6%. (E). Graph showing human fibroblast viability after 4, 12 and 24 h of incubation (1 × 10^6^ cells/well in complete MEM) of the HEC/Ag nanoparticle scaffolds at different AgNO3 concentrations. Reproduced with permission from 186. Copyright 2017, Elsevier.

The porosity of metal nanoparticle-hybrid hydrogels ensures stable exchange of water, oxygen, and metabolites at wound sites. Additionally, they can simulate the ECM in vitro, promoting cell attachment, proliferation, and differentiation. The Au nanoparticle rod/gelMA/alginate hydrogel provides an ideal 3D cultivation environment for cardiac muscle cells, improving cell adhesion and intercellular communication. As depicted in Figure 6, cardiac muscle cells cultured on the Au nanoparticle rod/gelMA/alginate hydrogel develop increased maturity and corresponding phenotypic structures after 7 days 58. Compared to pure GelMA, these cardiac muscle cells in the Au nanoparticle rod/GelMA/alginate hydrogel exhibit rhythmic contractions 58. Research has also shown that introducing gold nanoparticles into hydrogels can enhance the proliferation, vitality, and osteogenic differentiation of adipose-derived stem cells (ADSCs) and localize these effects over an extended period 197. Metal nanoparticles can also enhance the regenerative properties of hydrogels. For example, the incorporation of copper nano-based metal-organic framework nanoenzymes into hydrogels has been found to effectively improve oxidative stress and inflammation, providing a suitable microenvironment for the regeneration of osteochondral defects 198. In another study, magnetic Fe3O4 nanoparticles were loaded into chitosan/PEG hydrogel, resulting in higher viability and osteogenic differentiation ability of mesenchymal stem cells 199.

In addition to simulating the ECM and promoting cell migration and proliferation, an ideal artificial skin should possess excellent antibacterial and anti-inflammatory properties. This aligns well with the strengths of metal nanoparticle-hybrid hydrogel composites. Zulkifli *et al*. reported the fabrication of a low-toxicity hydroxyethyl cellulose/silver nanoparticle (HEC/Ag-nanoparticle) nanocomposite hydrogel scaffold that combines antibacterial, anti-inflammatory, and tissue regeneration properties, making it suitable for skin tissue engineering applications (Figure 6) 186. Rakhshaei *et al*. synthesized a chitosan-gelatin/ZnO nanoparticle composite hydrogel scaffold (CS-GEL/nZnO) in situ. This material exhibited good compatibility with normal human dermal fibroblasts, showing higher antibacterial efficacy and lower cytotoxicity 187, making it an ideal material for skin tissue engineering.

In conclusion, metal nanoparticle-hybrid hydrogels enhance the conductivity and mechanical strength of hydrogels, maintain a sterile environment, promote the in vitro expression of cellular phenotypes and increase maturity. They hold significant research prospects in the field of tissue-engineered skin construction. However, further research is needed to determine the fate of metal nanoparticles after hydrogel degradation.

## 6. Summary and prospects

Metal nanoparticle hybrid hydrogels epitomize a multifunctional wound care adjunct characterized by commendable mechanical attributes and environmental responsivity. This amalgamation enables precision and intelligent therapy and monitoring of wounds. This review encapsulates the structural variants, design strategies, and therapeutic applications of metallic nanostructured composite hydrogel materials in the treatment of acute and chronic wounds. It deliberates on how these materials engender novel or refined functionalities and elucidates how contemporary wound management harnesses these attributes. Furthermore, within the ambit of this evolving realm, it underscores the latent potential of metallic nanostructured composite hydrogel materials in revolutionizing wound care 7,22,32,71,81,200.

**Figure 7:**
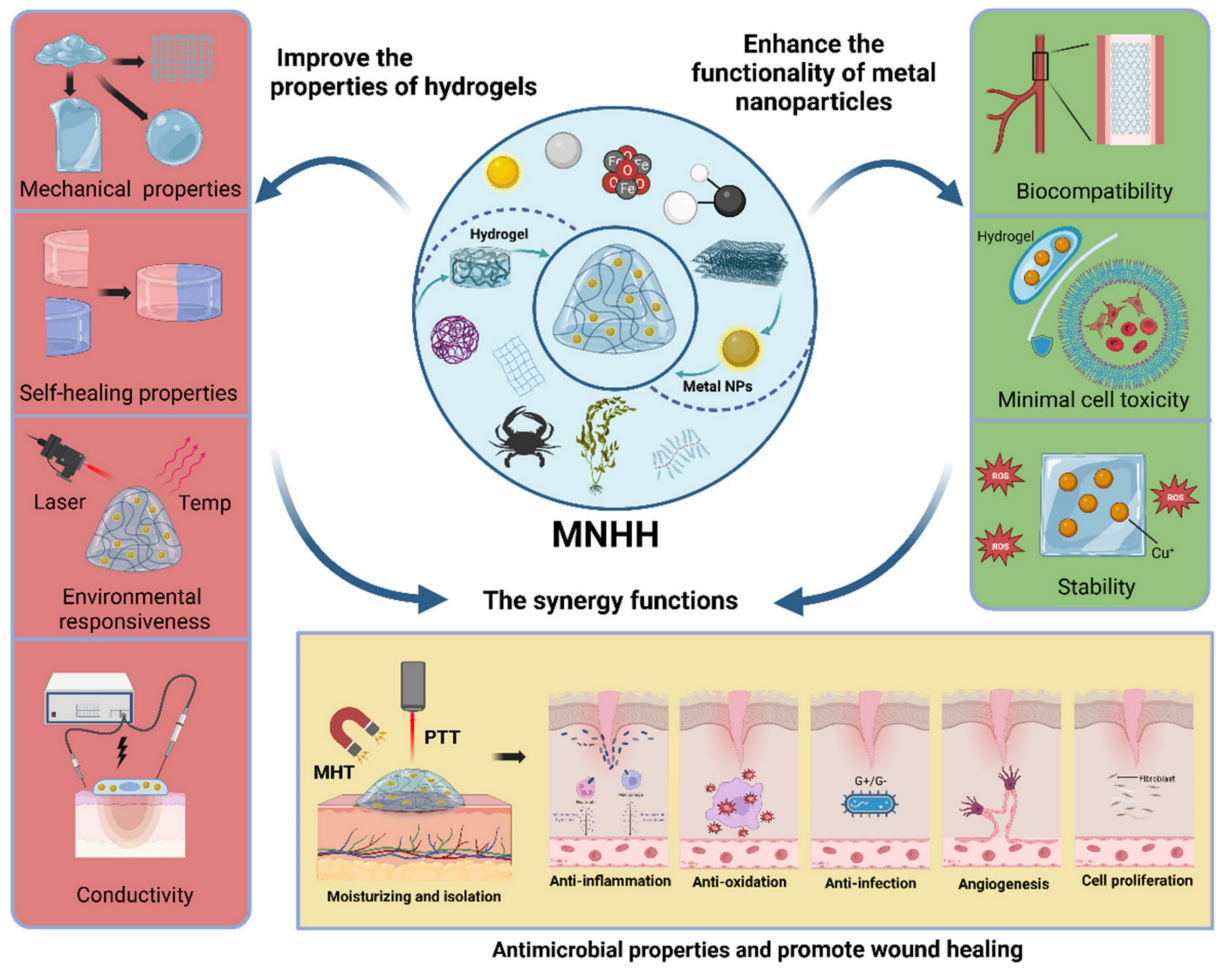
Illustration of MNHH combining the properties of metal nanocarriers with hydrogels. Metal nanoparticles can effectively improve the mechanical properties, self-healing properties, and electrical conductivity of hydrogels. Metal nanonanomaterials can also endow hydrogels with new environmental responsiveness. Hydrogels can stabilize metal nanoparticles and reduce their cytotoxicity. The combination of hydrogels and metal nanoparticles not only compensates for each other's deficiencies but also generates a powerful synergistic effect, such as synergistic antibacterial and wound healing properties. (Created with BioRender.com).

The amalgamation of metallic nanoparticles with hydrogels amalgamates their respective characteristics, blending strengths and mitigating weaknesses (Figure 7). This convergence not only augments the inherent attributes of metallic nanoparticles and hydrogels but also rectifies their intrinsic deficiencies, thus bestowing novel functional aspects upon both. Initially, the traits of metallic nanoparticles are incorporated within the hydrogel matrix, rendering the composite material selectively responsive to stimuli such as acoustic, optical, and electromagnetic cues, thereby effectuating precise wound treatment and monitoring 44. Second, alterations in the environmentally sensitive structure of the hydrogel modify the optical, chemical, or electronic properties of embedded metallic nanoparticles, achieving both stable nanoparticles and intelligent drug release, along with environmental dynamic sensing and monitoring 32,87. Third, the precise spatial arrangement of metallic nanoparticles within the hydrogel matrix passively amplifies their properties, engendering enhanced attributes suitable for biomedical applications such as tissue engineering and wound healing 32. The integration of metallic nanoparticles effectively enhances the structure of hydrogels, elevating mechanical and self-healing properties and enhancing their processability and pliability, thus broadening the scope of hydrogel dressings in wound care. Additionally, composite materials enhance hydrogel drug delivery systems, achieving stable, tunable, and controlled drug release systems, providing more precise and intelligent drug therapy for wounds 14,15,22. Metallic nanoparticles introduce novel functional traits to hydrogels. These composites can respond not only to local environmental changes but also to external stimuli such as light, heat, and magnetism, enabling the construction of multifunctional composite hydrogels capable of autonomous sensing, real-time monitoring, and controlled intervention. Conversely, hydrogels offer a means to address these shortcomings of metal nanoparticles. First, hydrogels can alleviate the potential toxicity associated with metal nanoparticles. Furthermore, hydrogels can reduce hemolytic activity and minimize damage to normal tissues. Additionally, hydrogels can enhance the long-term stability of metal nanoparticles. These features endow MNHH with a wide array of captivating capabilities. They can be employed to construct multifunctional materials for wound management and develop intelligent wound dressing systems integrating real-time monitoring and on-demand therapy, thereby surpassing the limitations of traditional wound care. These materials can serve as scaffolds for cell differentiation, manipulation, and proliferation, as well as engendering antibacterial biomaterials capable of eradicating drug-resistant pathogens. With these new attributes, composite hydrogels transcend the realm of being mere dressings for combating infection and promoting wound healing. They metamorphose into intelligent and adjustable wound management interfaces, ushering in new perspectives for precise wound treatment. These composite materials effectively mitigate the historical limitations of metallic nanoparticles in wound therapy while harnessing their advantages to optimize wound healing.

**Figure 8:**
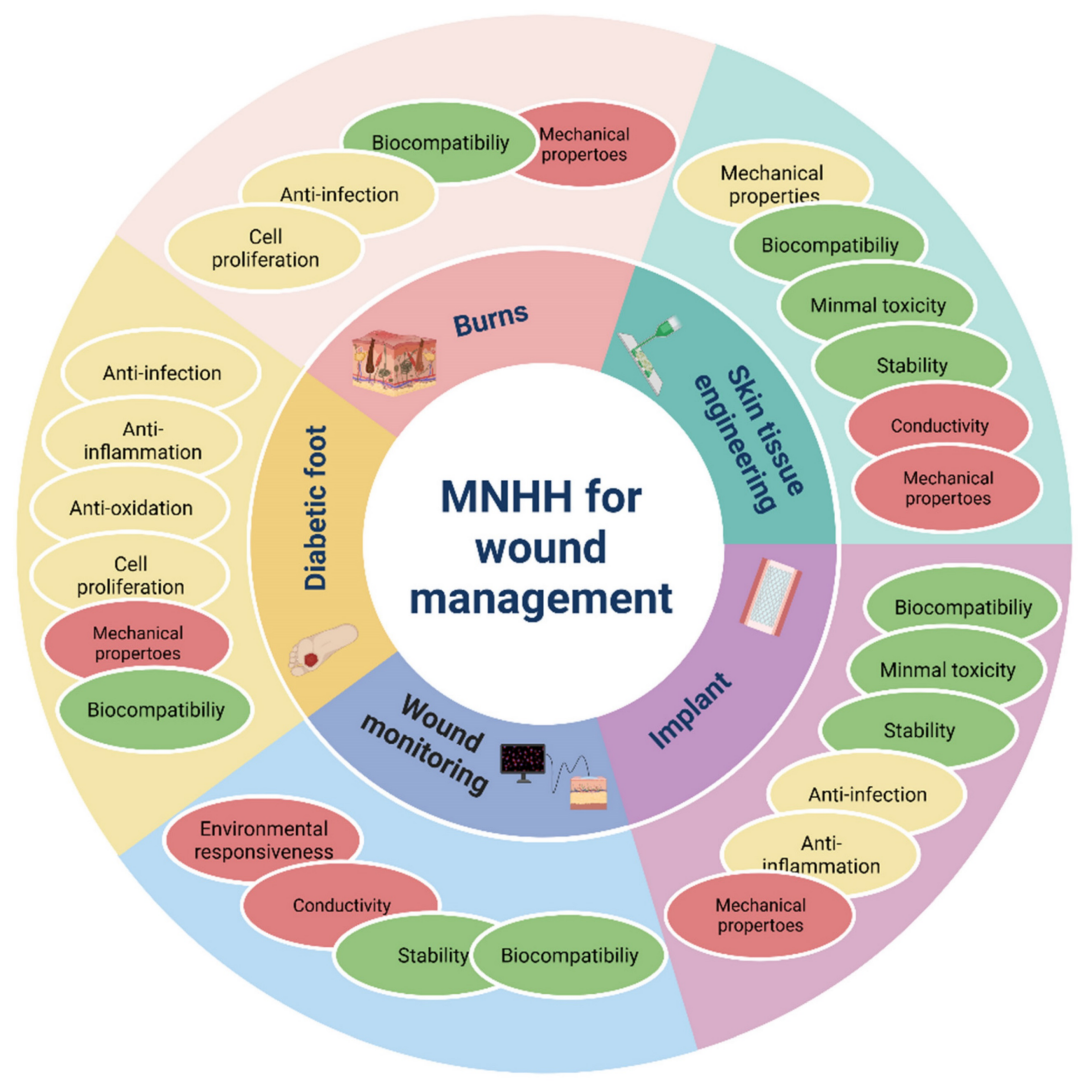
Mechanisms of MNHH for Wound Management. Metal nanoparticle hybrid hydrogels have excellent mechanical properties, plasticity, antibacterial, anti-inflammatory, environmental responsiveness, conductivity and stability, etc. These features can be used in various aspects of wound management, including treating acute wounds, treating chronic wounds, skin tissue engineering, medical implants, real-time monitoring of wounds, etc. (Created with BioRender.com).

Metal nanoparticle hybrid hydrogels represent an ideal material for wound management (Figure 8). Through structural modifications, composite materials enhance the mechanical properties and stability of hydrogels, endowing them with improved malleability and a broader scope of applications. Additionally, these composites amplify the resistance of hydrogels to infections and their capacity to expedite wound healing. In the context of acute wounds, metal nanoparticle hybrid hydrogels effectively absorb exudates from burn wounds while facilitating gas exchange. They exhibit exceptional bactericidal effects against various drug-resistant bacteria, effectively eradicating biofilms. Furthermore, these composites display significant hemostatic abilities, promoting wound contraction, collagen deposition, and blood vessel formation and reducing inflammation in infected burn wounds. They also mitigate the discomfort of frequent dressing changes. These hydrogels have anti-inflammatory and proangiogenic properties, promoting fibroblast migration and contributing to the regeneration of burn wound collagen, thereby expediting healing. For chronic wounds, composite hydrogels amalgamate the inherent antibacterial activity of metallic nanoparticles with photothermal and magneto-thermal effects, providing comprehensive, sustained, and precise antibacterial therapy for chronic infected wounds. They also effectively inhibit chronic wound biofilms. Moreover, composite hydrogels eliminate excessive ROS in wounds, maintain intracellular redox homeostasis, alleviate oxidative stress, and provide oxygen to improve the pathological microenvironment of diabetic wounds. Combined with attributes such as tissue adhesion, self-healing, injectability, and hemostasis, they stimulate cell proliferation and migration, along with the remodeling of the extracellular matrix, accelerating the healing of chronic wounds. In the realm of medical implants, metal nanoparticle hybrid hydrogels exhibit responsive release of antibacterial agents, ensuring long-term stable antibacterial effects while maintaining good tissue compatibility. They demonstrate no significant hemolytic or cytotoxic effects, reducing immune reactions and preventing the formation of collagen capsules. In the domain of tissue engineering, metal nanoparticle hybrid hydrogels can replicate the extracellular matrix (ECM) in vitro, offering an optimal 3D cultivation environment for cells. The presence of metallic nanoparticles enhances cell adhesion and communication, facilitating cell attachment, proliferation, and differentiation. With augmented swelling, biodegradation, and antibacterial abilities, they are relatively compatible with human dermal fibroblasts, encompassing characteristics such as low toxicity, antibacterial and anti-inflammatory properties, and the promotion of tissue regeneration. This provides novel pathways for the preparation of engineered skin tissues 194. Furthermore, these composite materials can undergo structural or color changes under specific environmental stimuli, responding to external triggers and sensing biological molecules or environmental shifts 192. This capability holds the potential to establish real-time monitoring of wound infections and enable on-demand drug administration in clinical settings 192. It also offers a robust reference for formulating wound treatment strategies that involve selective drug release 192. By embedding various forms of metallic nanoparticles within hydrogels, composite materials not only enhance the existing wound healing functions of both components but also generate unique attributes that a single material cannot provide. This innovation allows for real-time wound monitoring, precise drug administration, and intelligent responsiveness. This signifies that metal nanoparticle hybrid hydrogels are no longer merely wound healing dressings but have the potential to evolve into multifunctional tools for comprehensive wound management.

Hydrogels can be incorporated with various types of metal nanoparticles, such as monometallic nanoparticles, metal oxide nanoparticles, and alloys, each imparting distinct properties to the hydrogels, such as antimicrobial effects, environmental responsiveness, and improved mechanical properties. It is important to note that while the use of hydrogels can reduce the accumulation and toxicity of metallic nanoparticles in tissues, there is still a risk of these nanoparticles accumulating in organs such as the skin, liver, and kidneys over extended periods, potentially leading to various known or unknown toxic effects 95. Additionally, the preparation, application, and handling of metal nanoparticle hybrid hydrogels unavoidably release these nanoparticles into the environment, where they can accumulate in soil, water ecosystems, and living organisms, posing risks to ecological environments and human health. Further experiments and research are necessary to comprehensively investigate the long-term effects of metal nanoparticle hybrid hydrogels on both the human body and the environment.

By combining the biocompatibility of hydrogels with the conductivity of metallic nanoparticles, they can regulate cellular proliferation and migration, mimicking the extracellular matrix (ECM), thereby introducing novel avenues for engineering artificial skin tissues 194. Through the incorporation of diverse forms of metallic nanoparticles within hydrogels, a unique set of characteristics emerges, enhancing their existing wound-healing capabilities. This amalgamation allows for real-time monitoring, precision drug delivery, and intelligent responses to wound conditions. Capitalizing on comprehensive properties encompassing mechanics, chemistry, environmental responsiveness, and electrical performance, metal nanoparticle hybrid hydrogels bridge the gap between human tissues and conventional electronic devices, potentially revolutionizing the field of bioelectronics. In the visible future, tissue-engineered skin, versatile wound dressings, and intelligent wound management devices founded on such materials hold promising prospects for robust development.

## Figures and Tables

**Figure 1:**
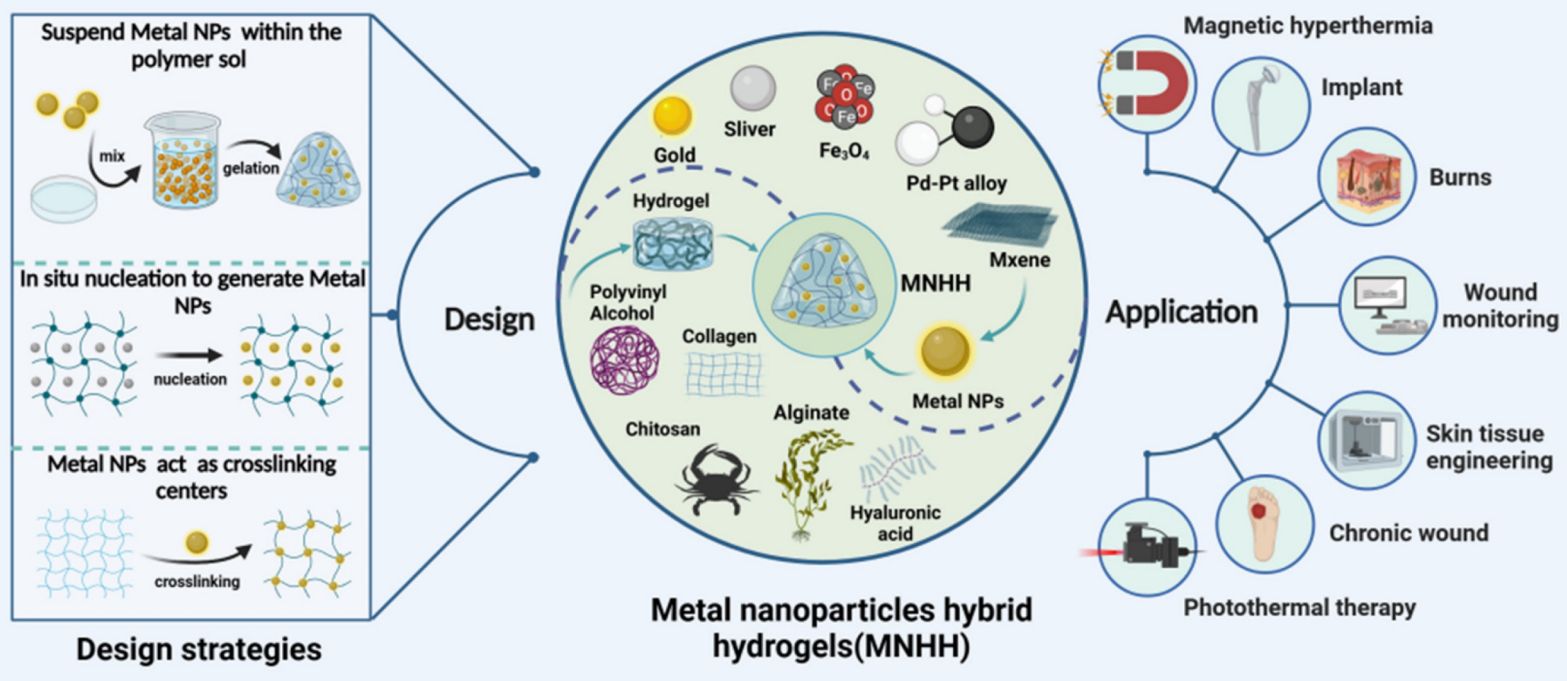
The design strategies and applications of MNHH. MNHH combining the advantages of hydrogel and metal nano have been widely used in the fields of wound healing. (Created with BioRender.com).

**Table 1:** The mechanisms by which the enhancement functions of MNNH operate

The enhancement function	Types of hydrogels	Specific performance	Refs.
Mechanical properties of hydrogels	SSG-TiO2 hydrogels	It reduced the zeta potential of SSG-TiO2 hydrogel, increased its density (from 47.1 g/ml to 50.15 g/ml), and particle size (from 277 nm to 764 nm), altering its surface tension.	59
Mechanical properties of hydrogels	Gelatin/CS/Ag hydrogels	The Young's modulus increased from 1.89 ± 0.62 MPa to 2.36 ± 0.54 MPa; the tensile strength increased from 1.62 ± 0.56 MPa to 1.92 ± 0.06 MPa; the fracture elongation (100%) increased from 29.23 ± 2.11 to 37.27 ± 1.65.	60
Mechanical properties of hydrogels	Cobalt nanoparticle magnetic crosslinked pHEMA hydrogel	Enhancing the thermal stability of the hydrogel, the ignition point increased from 228°C to 420°C; improving mechanical stability and flexibility, it can be stretched to 123% of its initial length.	61
Self-healing properties of hydrogels	Gel-DA@Ag hydrogel	The hydrogel is capable of self-healing within 5 minutes; when subjected to alternating high (500%) and low (1%) strains with amplitude oscillations, the hydrogel demonstrates a reversible gel-sol-gel transition, indicating a remarkable self-healing ability.	65
Stability and durability of drug release	AuC-liposome polyacrylamide hydrogel	Carboxyl-modified gold nanoparticles were employed as stabilizers for cationic liposomes, enabling the hydrogel to steadily release AuC-loaded liposomes over a span of 7 days.	71
Controlled release	PNAm-PDAAu-DOX hydrogel	Hydrogels exhibit the capability of mediating photothermal effects and controlled DOX release through intermittent near-infrared light irradiation.	68
Enlargement of drug release	layer-by-layer gold nanoparticle-loaded PDDA hydrogels	Under the stimulation of a magnetic field within the range of 40 to 110 Hz, the drug release rate from the hydrogel accelerates.	72
Conductivity of hydrogels	CS-GNP hydrogels	The incorporation of gold nanoparticles (GNPs) imparts electrical conductivity to the hydrogel, promoting active metabolism, migration, and proliferation of MSCs (mesenchymal stem cells).	73
Environmental responsiveness	Au@pNIPAM hydrogels	The formation of Au nanoparticle carriers results in distinct sensitivities and spatial resolutions, enabling the quantification of pyocyanin and allowing its spatial distribution within the biofilm to be imaged on the SERRS (surface-enhanced resonance Raman scattering) composite. schematic representation of wound contraction.	41
Environmental responsiveness	Au nanoparticles within dT-DMAEMA/HEMA hydrogels	The incorporation of gold nanoparticle carriers imparts pH-responsive electrochemical characteristics to the hydrogel: as the pH shifts from acidic to neutral, the hydrogel swells, increasing interparticle spacing and decreasing the conductivity of the composite, allowing for precise detection of pH changes.	81
Biocompatibility of the metal nanoparticles	Dex-G5-Ag hydrogel	G5 dendritic polymers exhibit pH-responsive Ag release and mitigate Ag tissue toxicity: When the gel is immersed in a pH 5.0 buffer solution, approximately 17.6% of Ag is released from the gel within 24 hours, whereas when immersed in a pH 7.4 buffer solution, this value decreases to only 6%.	82
Biocompatibility of the metal nanoparticles	Ag-PEG- Heparin hydrogel	A multilayer hydrogel system can mitigate Ag hemolytic activity and reduce damage to normal tissues.	83
Long-term stability	Cu NPs-Starch hydrogel	Copper nanoparticles (Cu NPs) are encapsulated within the hydrogel matrix, safeguarding Cu NPs from the influence of reactive oxygen species (ROS) while preserving their antibacterial activity.	84

**Table 2:** Hybrid hydrogels loaded with different kinds of metal nanoparticles

Metal Nano Types		Hydrogel Types	Functions	Refs.
Noble metal	Ag	SA/gelatin hydrogel; Polyacrylamide (PAM)/Polyvinyl alcohol (PVA); Composite alginate hydrogel; Chitosan-polyethylene glycol (PEG) hydrogel.	Mechanical properties/stability/biocompatibility/antibacterial performance: Ensured the stability of silver nanoparticles; Significantly enhanced the mechanical properties of the hydrogel; Robust antibacterial performance; Improved tissue compatibility.	90,91,95,97-101,148
	Au	Chitosan hydrogel; Poly(N-isopropylacrylamide) (PNIPAm) hydrogel; Soy protein hydrogel.	Mechanical properties/conductivity/environmental responsiveness/antibacterial performance: Amplified the antibacterial performance of the hydrogel; Enhanced the mechanical strength of the hydrogel; Elevated the hydrogel's conductivity, facilitating intercellular signal transmission; Augmented NANOG/CD34 expression, promoting wound healing; Imparted photothermal therapy (PTT) antibacterial effect to the hydrogel.	74,109-115
	Pt	PEG hydrogel; PVP hydrogel; OHA-CMCS hydrogel; Polyaniline hydrogel.	Conductivity/environmental responsiveness/antibacterial performance: Enhanced the photothermal therapy (PTT) effect of the hydrogel, leading to microbial eradication; Provided catalase activity to improve wound oxygenation; Offered conductivity, serving as a biosensor.	93,149-151
	Pd	Agarose-chitosan hydrogel;4arm-PEG-thiol hydrogel.	Stability/environmental responsiveness/antibacterial performance: Nanomaterials utilized as crosslinking agents to enhance the stability of hydrogels; Augmented PTT/PDT effects for bacterial and tumor cell eradication; Precisely controlled drug release.	152,153
Metal oxides	Iron oxide	PNIPAM hydrogel; paeonol-aldehyde (PA) and quaternized chitosan (QCS) hydrogel; PANI-P (AAm-co-AA) hydrogel.	Mechanical properties/conductivity/environmental responsiveness/antibacterial performance: Impart magnetic responsiveness to hydrogels, enabling effective penetration through wound biofilms; enhance photothermal therapy (PTT) effect for controlled drug release; create dual dynamic bond crosslinks to bolster mechanical properties of hydrogels; provide conductivity for use as a biosensor; mediate hydroxyl radical generation through catalytic degradation of hydrogen peroxide (CDT), leading to tumor cell destruction.	117,121,135,154-156
	Zinc oxide	Chitosan hydrogel; Alginate hydrogel.	Mechanical properties/biocompatibility/antibacterial performance: Enhance the antimicrobial efficacy, bioreactivity, and mechanical properties of composite materials; promote tissue regeneration.	120,121,157,158
	Copper oxide	Polyvinyl alcohol (PVA) hydrogel; Carboxymethyl cellulose hydrogel; Starch hydrogel; Gelatin modified with methyl acrylate (Gel-MA) hydrogel; CPAP/PDA hydrogel.	Self-healing properties/environmental responsiveness/antibacterial performance: Improve the self-healing capability of hydrogels; boost photothermal therapy (PTT) performance of hydrogels; stimulate fibroblast proliferation; enhance hydrogel's antibacterial potential, effectively targeting fungi.	118,122-125,127,128,159,160
	Manganese oxide	PEGMA-co-GMA-co-AAm (PPGA) hydrogel; HA-ADH hydrogel; Starch hydrogel; Pluronic F127 hydrogel; Silk fibroin (SF)/Carboxymethyl cellulose (CMC) hydrogel; Polydopamine (DSAMP) hydrogel.	Environmental responsiveness/lipase-like activity/antibacterial performance: Clear ROS, generate oxygen, modulate wound microenvironment, alleviate inflammation; reinforce hydrogel's antibacterial and antifungal properties; endow hydrogel with photocatalytic capabilities (PTT); confer peroxidase activity to hydrogel.	136,161-164
polymetallic	Ag+Au	Chitosan hydrogel; iota carrageenan (CA)- poloxamer 407 (F127) hydrogel.	Mechanical properties/environmental responsiveness/biocompatibility/antibacterial performance: Enhance hydrogel mechanical properties; improve silver ion release, reduce cytotoxicity of silver nanoparticles; exhibit excellent biocompatibility and near-infrared (NIR) photothermal responsiveness.	139,141,165-167
	Au/Ag+ZnO	Carboxymethyl cellulose (CMC) hydrogel; polyethylene glycol (PEG) hydrogel.	Stability/environmental responsiveness/biocompatibility/antibacterial performance: Enhance the antimicrobial capability of hydrogels; reduce the cytotoxicity of gold/silver nanoparticles; maintain the stability of metal nanoparticles and PDT performance.	140,145,165,168-170
	Au+Pt	Oxidized hyaluronic acid (OHA)/carboxymethyl chitosan (CMCS) hydrogel; hyaluronic acid hydrogel; polydopamine (PDA) hydrogel.	Environmental responsiveness/lipase-like activity: Provide glucose oxidase and catalase activity to improve the diabetic wound microenvironment; enhance photothermal conversion efficiency (PCE) up to 81.78%; eliminate the ROS generated as byproducts during the PTT process.	138,171-173
	Ag+ Fe_3_O_4_	Starch-based hydrogel.	Environmental responsiveness/antibacterial performance: Hybrid hydrogels exhibit magnetic responsiveness, facilitating the penetration of silver nanoparticles through biofilms and enhancing antibacterial efficacy.	144,171,174,175
	Pt+Pd	PEG hydrogel; HA hydrogel.	Environmental responsiveness/lipase-like activity: Imparting catalase (CAT)-like and peroxidase (POD)-like activities, as well as photothermal therapy (PTT) capability, to the hydrogel.	176,177

**Table 3:** Application of metal nanoparticle hybrid hydrogels in the field of wound healing.

Application	Hydrogel Types	Therapeutic Effects	Refs.
Chronic Wounds	Chitosan-PEG-Silver Nitrate-Based Hydrogel	The hydrogel ensures a continuous and stable release of silver ions, effectively combating bacterial infections. The silver nanoparticle-infused hydrogel exhibits higher porosity, increased swelling capacity, and elevated water vapor transmission rate (WVTR). Furthermore, it enhances antioxidant properties and promotes the healing of diabetic rabbit wounds.	56
	QCT-Ag NPs-Carbopol 934 hydrogel	Enhanced effectiveness against *Staphylococcus aureus* and *Escherichia coli*; Increased percentage of epithelialization in diabetic wound healing.	178
	Au-Pt-OHC hydrogel	Metal nanoparticles in synergy with carboxymethyl chitosan (CMCS) exhibit antibacterial properties, protecting the wound site; Metal nanoparticles possess similar activities to glucose oxidase (GOD) and catalase (CAT), which can lower blood sugar, reduce oxidative damage, provide oxygen, improve the pathological microenvironment of diabetic wounds, and facilitate wound healing; The dynamic cross-linking through Schiff base reaction imparts excellent self-healing properties to the OHCN hydrogel dressings.	138
	PB@PDA@Ag hydrogel	The hydrogel demonstrates excellent photothermal therapy (PTT) performance, capable of disrupting cell integrity under laser irradiation, generating reactive oxygen species (ROS), and reducing ATP and oxidized GSH to synergistically combat MRSA. Simultaneously, the hydrogel mitigates local inflammatory responses and upregulates wound VEGF expression, effectively accelerating the healing of diabetic wounds infected with MRSA.	97
	MnO2-silk fibroin (SF)-carboxymethyl cellulose (CMC) hydrogel	The hydrogel exhibits injectability, suitable for irregular wounds in diabetes; encapsulated MnO2 nanoparticles can catalyze excessive ROS, alleviate wound oxidative stress, promote angiogenesis, and reduce inflammation levels; the hydrogel can counteract the overexpression of matrix metalloproteinases (MMPs) in diabetic wounds (over 80%) and facilitate extracellular matrix remodeling; the nanocomposite hydrogel accelerates diabetes wound healing, with wound healing rates >76% in 7 days and 100% in 14 days.	161
	(HA-DA)-PDA-Ti_3_C_2_ MXene hydrogel	The HA-DA molecules regulate macrophage polarization from M1 to M2 to achieve anti-inflammatory effects; the hydrogel exhibits mild PTT capabilities and controlled oxygen release; it clears excessive ROS from wounds, maintaining intracellular redox homeostasis and alleviating oxidative stress; possessing multifunctionality such as tissue adhesion, self-healing, injectability, and hemostasis, combined with gentle photothermal stimulation, greatly promotes proliferation and migration of human umbilical vein endothelial cells, facilitating the healing of infected diabetic wounds.	179
Acute Wounds	Collagen (GT)-Ag NPs hydrogel	The GT/Ag freeze-dried hydrogel exhibits a swelling ratio of up to 4000%, effectively absorbing exudate from burn wounds and allowing gas exchange. It demonstrates excellent bactericidal effects against methicillin-resistant *Staphylococcus aureus* (MRSA) and *Pseudomonas aeruginosa* (PA), effectively removing biofilms. The hydrogel also possesses remarkable hemostatic capability. It promotes contraction of infected burn wounds, deposition of collagen protein, and angiogenesis while reducing inflammation. The hydrogel maintains stability in wound environment and degrades at a moderate rate (4 weeks), minimizing the discomfort of frequent dressing changes.	180
	Human amniotic membrane (HAM)- Ag NPs- carbopol 934 hydrogel	The hydrogel exhibits antibacterial activity against both gram-positive and gram-negative bacteria. It possesses anti-inflammatory properties, stimulates angiogenesis, and promotes fibroblast migration. It significantly accelerates burn wound healing, as evidenced by a wound contraction percentage of 96.1 ± 0.27% after 20 days (p < 0.0001) and an epithelialization period of 23.67 ± 2.05 days (p < 0.01).	181
	Silver nanoparticle-thermosensitive methylcellulose (MC) hydrogel	Exhibiting excellent antibacterial activity against a variety of bacteria by 99.9%, effectively promoting collagen regeneration in burn wound surfaces, and accelerating the healing process.	182
Implants	Dex-G5-Ag hydrogel	The hydrogel exhibits pH-responsive release of antibacterial agents, displaying effective antibacterial activity against both gram-negative bacteria (*Escherichia coli* and *Pseudomonas aeruginosa*) and gram-positive bacteria (*Staphylococcus epidermidis* and *Staphylococcus aureus*); no significant hemolytic toxicity, cytotoxicity, tissue, or biochemical toxicity is observed upon cell incubation or implantation.	183
	A poly (hydroxyethyl methacrylate)-Ag nanoparticle porous hydrogel	The hybrid hydrogel exhibits robust antibacterial efficacy against both gram-positive bacteria (*Staphylococcus aureus*) and gram-negative bacteria (*Escherichia coli*). Additionally, it demonstrates diminished immune response, showcasing in vivo resistance to foreign body reaction (FBR), effectively preventing the formation of collagenous capsules.	184
	Ag-starPEG-heparin hydrogel	The hydrogel maintains a prolonged antibacterial effect against strains of *Escherichia coli* and *Staphylococcus epidermidis*. Simultaneously, it demonstrates excellent hemocompatibility, with no evident hemolytic effects observed.	185
Tissue Engineering	Gold nanorod/GelMA/alginate hydrogel	Hydrogels can mimic the extracellular matrix (ECM) in vitro, providing cells with an ideal 3D culture environment. The presence of gold nanorods enhances cell adhesion and intercellular communication, promoting cell attachment, proliferation, and differentiation.	58,73
	Hydroxyethyl cellulose (HEC) combined with silver nanoparticles (Ag NPs)	The composite material combines the characteristics of low toxicity, antibacterial, anti-inflammatory, and tissue regeneration promotion, making it highly suitable for applications in skin tissue engineering.	186
	Chitosan-gelatin/ZnO nanocomposite hydrogel scaffold (CS-GEL/nZnO)	The hydrogel exhibits a high porosity (pore size ranging from 50-400μm) with well-distributed ZnO. It demonstrates enhanced swelling, biodegradability, and antibacterial properties. It can effectively control drug release and shows good compatibility with normal human skin fibroblasts. In situ synthesis of ZnO enhances its antibacterial efficacy and reduces cytotoxicity, making it an ideal material for skin tissue engineering.	187

## Abbreviations

ADSCs: adipose-derived stem cells
Ag NPs: silver nanoparticles
Cu NPs: copper nanoparticles
CMCS: carboxymethyl chitosan
DFUs: diabetic foot ulcers
DOX: doxorubicin
DMAEMA: dimethyl aminoethyl methacrylate
ECM: extracellular matrix
GT: gelatin
HCG: human chorionic gonadotropin
HEMA: hydroxyethyl methacrylate
LSPR: local surface plasmon resonance
MNHH: metal nanoparticle hybrid hydrogels
MRSA: methicillin-resistant Staphylococcus aureus
MSCs: mesenchymal stem cells
MC: methylcellulose
NCs: nanocomposites
OHA: oxidized hyaluronic acid
PDA: polydopamine
PVA: polyvinyl alcohol
PEG: polyethylene glycol
PA: pseudomonas aeruginosa
ROS: reactive oxygen species
SERRS: surface-enhanced resonance Raman scattering
SPIONs: superparamagnetic iron oxide nanoparticles
